# Large Amino Acid Mimicking Selenium-Doped Carbon Quantum Dots for Multi-Target Therapy of Alzheimer’s Disease

**DOI:** 10.3389/fphar.2021.778613

**Published:** 2021-10-27

**Authors:** Xi Zhou, Shuyang Hu, Shuangling Wang, Yu Pang, Yulong Lin, Meng Li

**Affiliations:** College of Pharmacy, Key Laboratory of Innovative Drug Development and Evaluation, Hebei Medical University, Shijiazhuang, China

**Keywords:** alzheimer’s disease, selenium-doped carbon quantum dots, amyloid β peptides, peptide aggregation, anti-oxidant activity, multi-target therapy

## Abstract

Multi-target intervention and synergistic treatment are critical for the drug development of Alzheimer’s disease (AD) due to its complex and multifactional nature. Oxidative stress and amyloid β peptides (Aβ) accumulation have been recognized as therapeutic targets for AD. Herein, with ability to inhibit Aβ aggregation and the broad-spectrum antioxidant properties, the large amino acid mimicking selenium-doped carbon quantum dots (SeCQDs) are presented as novel nanoagents for multi-target therapy of AD. Compared with the precursor, selenocystine, SeCQDs which maintain the intrinsic properties of both selenium and carbon quantum dots (CQDs) possess good biocompatibility and a remarkable ROS-scavenging activity. Moreover, the functionalized α-carboxyl and amino groups on edge of SeCQDs can trigger multivalent interactions with Aβ, leading to the ability of SeCQDs to inhibit Aβ aggregation. *In vivo* study demonstrated that SeCQDs can significantly ameliorate the Aβ induced memory deficits, reduce Aβ accumulation and inhibit neuron degeneration in AD model rats. The versatility of functionalization and potential ability to cross the blood-brain barrier (BBB) make SeCQDs as prospective nanodrugs for treating AD.

## Introduction

Alzheimer’s disease (AD) as one of the most prevalent types of dementia has been reported to affect approximately 10% of people aged 65 years or more (Palop and Mucke, 2010; Zhang et al., 2019; Kim et al., 2020). The main pathological features of AD are the accumulation of extracellular plaques consisting of amyloid β peptides (Aβ) and the formation of neurofibrillary tangles which are composed of hyperphosphorylated tau filaments in the brain (Palop and Mucke, 2010; Zhang et al., 2019; Kim et al., 2020). Although the exact pathological mechanism of AD remains to be elucidated, a significant body of evidence has demonstrated the existence of cross-talk between Aβ deposition and neurodegeneration in AD (Palop and Mucke, 2010; Zhang et al., 2019; Kim et al., 2020). Assembly of Aβ into soluble oligomers and subsequent aggregates plays a critical role in the pathogenesis of AD (Zhang et al., 2019). The aggregates-induced dysfunction has been known to be a possible cause of AD through various molecular signaling pathways including abnormal production of reactive oxygen species (ROS), which will trigger a series of damages of cellular components and lead to the oxidative stress in AD (Lei et al., 2021). Additionally, the oxidative stress in turn promotes the accumulation of Aβ (Eric and Eugenia, 2017). Thus, inhibiting Aβ aggregation and the formation of ROS is a reasonable and effective therapeutic strategy for AD.

To this aim, various small molecules with antioxidant activity or the ability to inhibit Aβ aggregation have been developed for AD treatment (Ono et al., 2006; Jokar et al., 2019; Iraji et al., 2020). However, the side-effects, limited efficacy especially the poor permeability of the blood-brain barrier (BBB) hindered their clinical use. To overcome these limitations, nowadays, nanomaterials as novel therapeutic agents have been designed to intervene in the pathology of AD due to their unique structural superiority, high stability and ready ability to cross the BBB (Du et al., 2021; Zeng et al., 2021). Therefore, with proper design, the nanosystems would treat nervous system diseases with efficacy superior to small-molecule drugs.

On the basis of this concept, many efforts have been recently devoted to design pharmaceutical nanomaterials for potential AD treatments, including metal nanoparticles, carbon-based nanostructures and polymeric nanomaterials (Zeng et al., 2021). Among these nanomaterials, selenium doped nanoparticles (SeNPs) have attracted great interest because of the fundamental significance of selenium in cellular redox regulation, detoxification, and protection of immune-system (Zhang et al., 2014; Menon et al., 2018; Liu et al., 2020). Although promising, most of these reported SeNPs were synthesized via chemical reduction methods. The use of toxic reducing and capping agents may hinder their biological applications. Importantly, it has been reported that increasing the size of nanoparticles can decrease the percentage of their brain accumulation and reduce their biological activities (Mahmoud et al., 2020). Thus, the relatively large size of these reported SeNPs make them not suitable for the treatment of nervous system diseases. Moreover, considering the complex pathogenetic mechanisms of AD, compared with these SeNPs only exerted antioxidant ability, selenium-based nanocomposites with multifunctional performance against AD would be more desirable.

**FIGURE 1:**
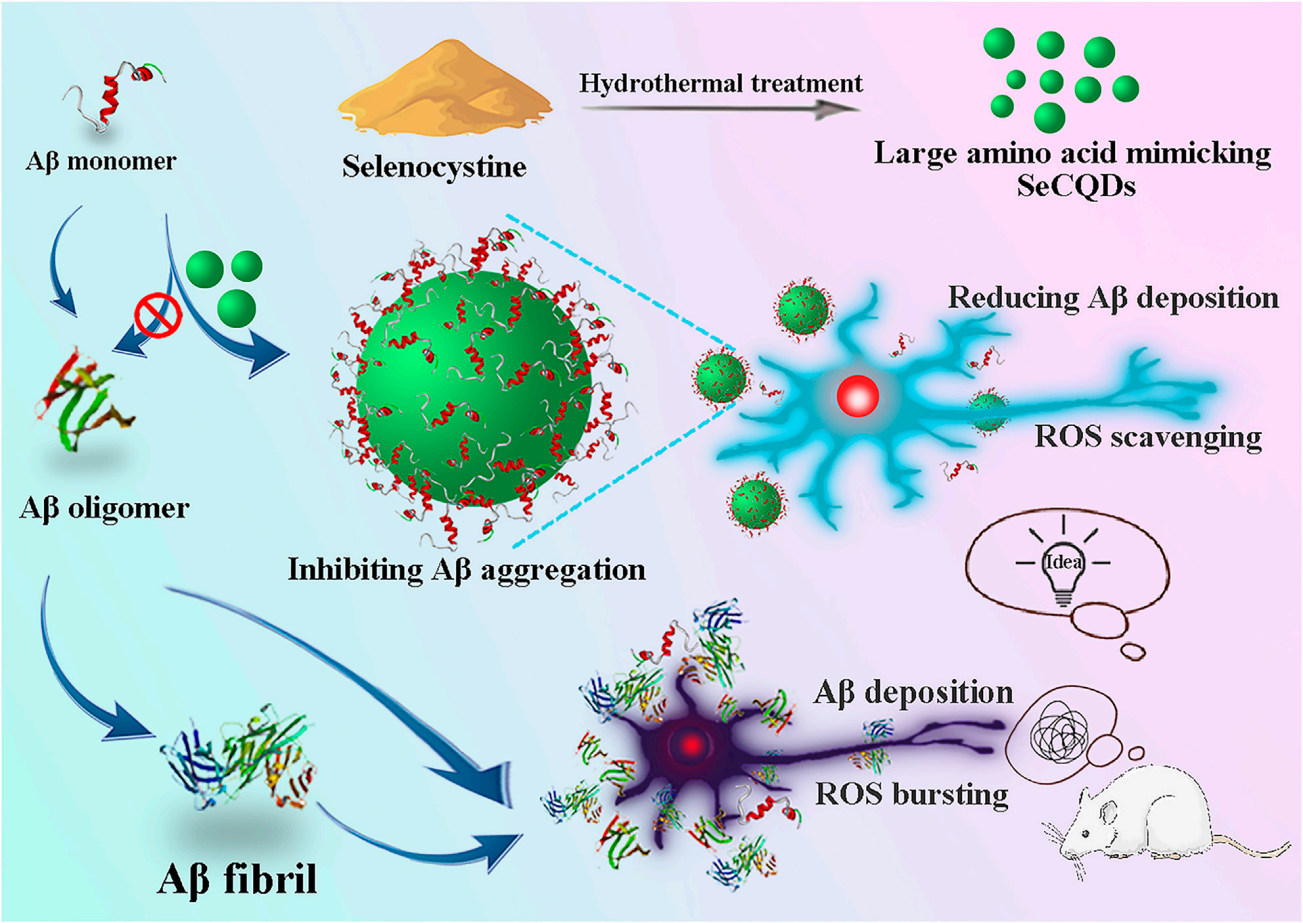
Synthesis of SeCQDs and illustration of the inhibition effects of SeCQDs on Aβ aggregation and ROS production as well as mitigation of potential neurotoxicity in AD rat model.

Towards the development of multifunctional selenium-based nanocomposites with small size and high biocompatibility for AD therapy, herein, selenium-doped carbon quantum dots (SeCQDs), have been rationally designed and successfully applied to not only inhibit Aβ aggregation but also scavenge the produced ROS in the brain. The SeCQDs were synthesized via the simple hydrothermal treatment of selenocystine (SeCys) (Li et al., 2017), one of the naturally occurring forms of selenium (Yu et al., 2015). Taking the advantages of the excellent biocompatibility, optical properties and easy to cross the BBB, carbon quantum dots (CQDs) have emerged as a promising class of imaging agents and drug agents for various biomedical applications, especially in diagnosis and treatment of neurological disorders (Yu et al., 2015; Devi et al., 2019; Ashrafizadeh et al., 2020). With selenium doping, the SeCQDs maintained the intrinsic properties of both selenium and CQDs. Critically, after calcination of SeCys, the obtained SeCQDs possess paired α-carboxyl and amino groups on their edge, which trigger multivalent interactions with Aβ. The large amino acid mimicking SeCQDs can ameliorate the Aβ induced memory deficits, reduce Aβ accumulation and inhibit neuron degeneration in AD model rats (Figure 1). Compared with the reported SeNPs which only exert antioxidant ability (Zhang et al., 2014; Rao et al., 2019), the multifunctional properties of the synthesized SeCQDs are essential for AD treatment since the pathogenetic mechanisms of AD is complex. This finding may open a new avenue for the design of multifunctional nanoagents for AD therapy.

## Materials and Methods

### Synthesis of SeCQDs

SeCQDs were fabricated by a hydrothermal treatment according to the previous report (Li et al., 2017). Briefly, SeCys (200 mg) was dissolved in deionized water (12 ml). Then the pH of the solution was adjusted to pH 8.5 by NaOH to make sure the sample was completely dissolved. The solution was transferred to a stainless autoclave and heated at 80°C for 24 h in an oven. After that, the suspension was centrifuged at 12,000 rpm and the supernatant was collected and dialyzed. Then brown powder was obtained after freeze-dry of the SeCQDs solution.

### Detection of Hydroxyl Radicals (•OH) via Electron Paramagnetic Resonance (EPR) Spectra

To detect •OH, 5,5-dimethyl-1-pyrroline-N-oxide (DMPO) was used as the spin trapping agent to trap •OH. A solution containing DMPO (100 mM), H_2_O_2_ (100 mM) and SeCQDs aqueous solution (5, 10, 50 μg ml^−1^) in PBS (10 mM, pH 7.0) in a quartz cuvette was exposed to UV light (365 nm, 60 mW cm^−2^) for 7 min. Then, the solution was transferred into a capillary tube, which was mounted onto the EPR spectrometer for scanning.

### Detection of Hydroxyl Radicals via Methylene Blue and Disodium Terephthalate Based Assay

The formation of •OH was also monitored by the colorimetric assay and fluorescent assay, in which methylene blue (MB) and terephthalate (TA) were utilized as the dyes, respectively. TA can react with •OH radicals to generate a highly fluorescent product, 2-hydroxyl terephthalic acid (TAOH). To detect •OH, 400 μl of PBS (pH 7.0) containing H_2_O_2_ (100 mM) and MB (20 μM) or TA (40 μM) was added to the SeCQDs solution (0, 5, 10, 50 μg ml^−1^) and exposed to UV light (365 nm, 60 mW cm^−2^) for 3 min. After that, the samples were centrifuged at 12,000 rpm for 5 min. The supernatants were collected for measurements.

### Thioflavin T Binding Fluorescence

Aβ40 peptides (100 μM) with or without various concentrations of SeCQDs or SeCys (5, 50 μg ml^−1^) were incubated at 37°C for 7 days in aggregation buffer. At different times, aliquots of each sample were taken for fluorescence measurements. The final concentration of Aβ40 used for measurements was kept at 1 μM, and the thioflavin T (ThT) concentration was 10 μM. The excitation wavelength was 444 nm, and the emission intensity at 482 nm was used for analysis.

### Transmission Electron Microscopy

The peptide samples (10 μl) were spotted onto carbon-coated copper grids and stained with 1.5% (w/v) phosphotungstic acid (pH 7.4). Grids were air-dried before analysis on the transmission electron microscopy (TEM).

### NMR Spectroscopy

Samples for NMR were run in aqueous HEPES buffer with 10% ^2^H_2_O added. Samples containing Aβ40 were run at 0.2 mM. SeCQDs were incubated with Aβ40 for 2 h at 37°C. NMR measurements were carried out on a Bruker 600-MHz AVANCE NMR spectrometer equipped with a triple channel cryoprobe at 5°C. The concentration of SeCQDs was 50 μg ml^−1^.

### Intracellular Detection of Reactive Oxygen Radicals

To prepare different peptide samples, Aβ40 peptides (100 μM) with or without various concentrations of SeCQDs (5, 50 μg ml^−1^) were incubated at 37°C for 7 days in aggregation buffer. The generation of ROS in PC12 cells (rat pheochromocytoma, American Type Culture Collection) was monitored using 2′,7′-dichlorofluorescein (DCF) diacetate (Beyotime, China). This nonfluorescent and cell-permeable dye can be converted to the anionic but nonfluorescent form DCFH by intracellular esterases. On the action of intracellular ROS, DCFH would be oxidized into its highly fluorescent form DCF, whose fluorescence intensity correlates with the amount of intracellular reactive oxygen radicals. To perform the test, PC12 cells which pretreated with different peptide samples for 12 h were incubated with 20 μM DCF diacetate for 60 min at 37°C. The cells were then rinsed with PBS solution. The fluorescence intensity was monitored on a fluorescence spectrofluorometer with excitation and emission wavelengths of 488 and 525 nm, respectively.

### Cell Toxicity Assays

PC12 cells were cultured in DMEM (Gibco BRL) medium supplemented with 5% fetal bovine serum (FBS), 10% horse serum (HS) in a 5% CO_2_ humidified environment at 37°C. Cells were plated at 7 000 cells per well on poly-l-Lysine coated 96-well plates in fresh medium. After 24 h, Aβ40 (10 μM) that had been aged with or without various concentrations of SeCQDs were dispensed into the PC12 cells, and the cells were further incubated for 24 h at 37°C. Cytotoxicity was measured by using MTT (3-(4,5-dimethylthiazol-2-yl)-2,5-diphenyltetrazolium bromide, Sigma-Aldrich) assay. Absorbance values of formazan were determined at 570 nm with a Bio-Rad model-680 microplate reader.

### Biodistribution and Biocompatibility Study

Seven-week-old male C57BL6/J mice with body weights between 18 and 20 g were obtained from the Experimental Animal Center of the Chinese Academy of Medical Sciences. All the protocols and procedures for animal handing were carried out following the guidelines of the Hebei committee for care and use of laboratory animals, and were approved by the Animal Experimentation Ethics Committee of the Hebei Medical University. After intravenous injection of 100 μl SeCQDs (1 mg ml^−1^), the main organs were collected after 6 h. The Se content of the samples was measured by ICP-MS (Agilent 7800 ICP-MS). The data points shown are the mean values ± SEM from three independent experiments (Three mice were used for each group.).

For the biocompatibility study, the main organs were collected and dissected to make paraffin section 14 days after i. v. injection of SeCQDs (75 μg per mouse). Then, Hematoxylin-eosin (H&E) staining assay was conducted.

### AD Rat Model Preparation and Treatment Procedures

Adult male SD rats (320 ± 30 g) were obtained from Laboratory Animal Resources, Chinese Academy of Sciences. All the protocols and procedures for animal handing were carried out following the guidelines of the Hebei committee for care and use of laboratory animals, and were approved by the Animal Experimentation Ethics Committee of the Hebei Medical University. The rats were housed under standard condition with a light exposure of 12 h light/12 h dark cycle and free access to food and water. The rats were denied to access to food for 12 h before the test. After that, they were anesthetized by intraperitoneal injection of sodium pentobarbital (45 mg kg^−1^). Aβ40 oligomers (1.0 mg ml^−1^, total volume of 10 μl, 2 μl min^−1^) were injected into each side of the hippocampus. The position of cornu ammonis area 1 (CA1) was obtained by subtracting 3.5 mm from the anteroposterior position, 2.0 mm from the mediolateral position, and 3.0 mm from the dorsoventral position. The experimental rats were housed for 3 weeks to establish the AD rat model. Aβ40 oligomers were freshly prepared by dissolving Aβ40 in 0.1% trifluoroacetic acid to give a concentration of 10 μg μl^−1^, followed by incubation for 7 days at 37°C. The rats in control groups were injected with saline instead of Aβ40 oligomers. In the treating group, SeCQDs (100 μg ml^−1^, total volume of 100 μl) or SeCys (100 μg ml^−1^, total volume of 100 μl) were administrated to AD model rats via tail vein injection every 2 days for a consecutive 21 days after the models had been established for 3 days.

## Results and Discussion

### Characterizations of SeCQDs

**FIGURE 2:**
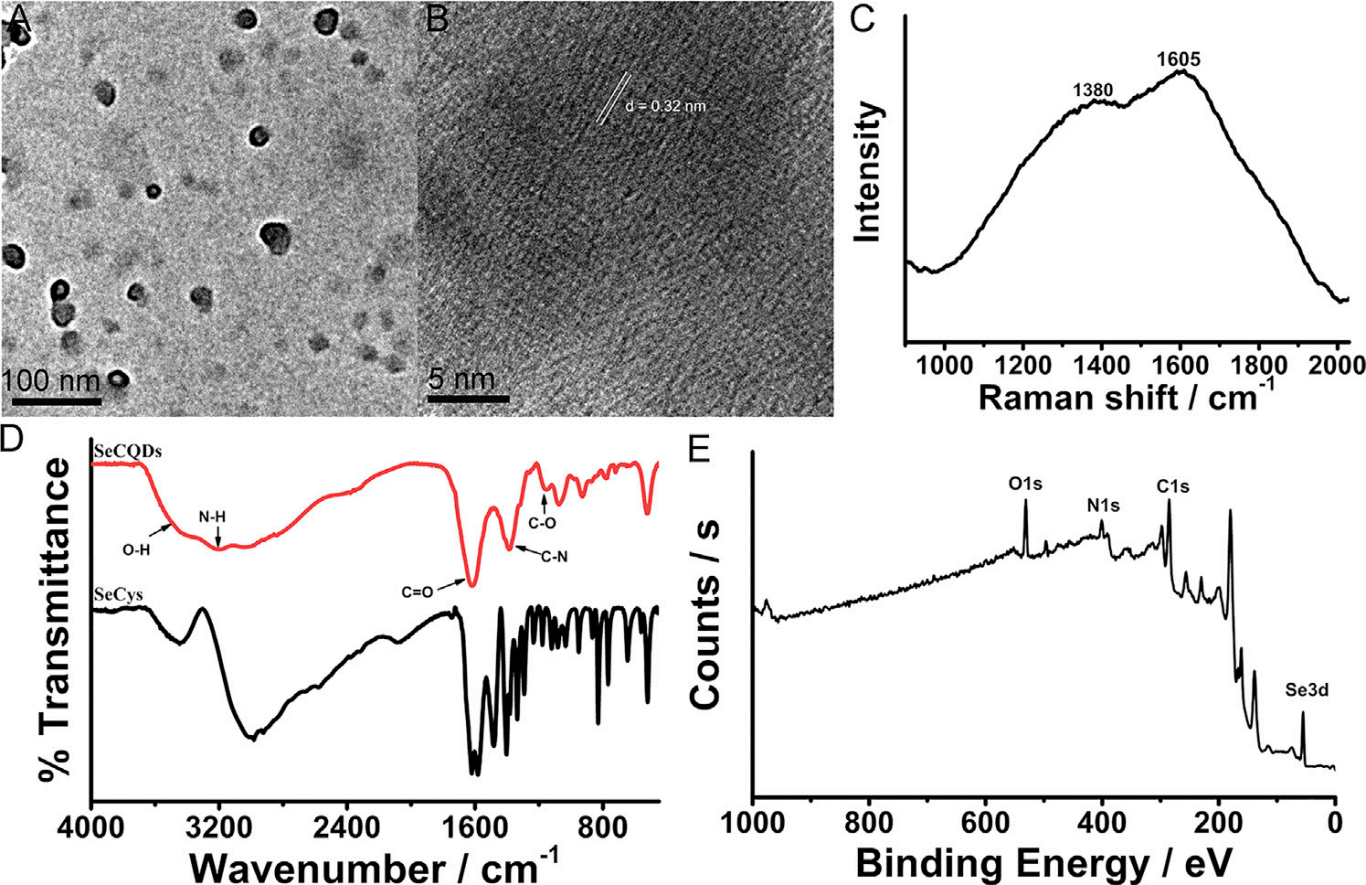
The characterization of the as-synthesized SeCQDs. TEM image of SeCQDs **(A)**. HRTEM image of SeCQDs **(B)**. Raman spectrum of SeCQDs **(C)**. FT-IR spectra of SeCQDs and SeCys **(D)**. XPS spectrum of SeCQDs **(E)**.

SeCQDs were synthesized by a hydrothermal treatment of SeCys according to the previous report (Li et al., 2017). The TEM images showed that SeCQDs were well dispersed with a diameter of approximately 25 nm (Figure 2A). A typical lattice spacing of 0.32 nm was clearly observed in the high-resolution TEM (HRTEM) images (Figure 2B), which was similar to the bulk graphite (002 facet) (Li et al., 2017; Rosenkrans et al., 2020). The peak in X-ray diffraction (XRD) pattern of SeCQDs at 22° was also assigned to the (002) plane (Supplementary Figure S1) (Rosenkrans et al., 2020). The relatively small size revealed the possibility of SeCQDs to penetrate the BBB and be used as agents for AD treatment. The UV-Vis absorption spectrum of SeCQDs showed two typical absorption peaks at approximately 280 and 340 nm due to the existence of multiple electron transitions (Supplementary Figure S2) (Li et al., 2017). The high degree of crystallinity was further confirmed by Raman spectrum analysis, in which the crystalline G band at 1,605 cm^−1^ was stronger than the disordered D band at 1,380 cm^−1^, with a G-to-D intensity ratio (IG/ID) of 1.2 (Figure 2C). Stretching vibrations for O-H or N-H (Bonds between 3,100 cm^−1^ and 3,600 cm^−1^), C=O (1,615 cm^−1^), C-N (1,381 cm^−1^) and C-O (1,154 cm^−1^) bonds were observed in Fourier translation infrared (FT-IR) spectrum (Figure 2D), demonstrating the formation of polyaromatic structures and the existence of free carboxyl and amino groups at the edge of SeCQDs. The composition of SeCQDs was also analyzed using X-ray photoelectron spectroscopy (XPS), which revealed that SeCQDs were primarily composed of carbon, oxygen, nitrogen, and selenium (Figure 2E, Supplementary Figure S3). In addition, the stability of SeCQDs in different solutions, including water, PBS (pH 7.4) and cell culture medium (DMEM supplemented with 10% fetal bovine serum) was also investigated since it was a key factor for their practical applications in biological systems. As shown in the dynamic light scattering (DLS) studies and TEM results, the particle size (Supplementary Figure S4A) and morphology (Supplementary Figure S4B-D) of SeCQDs did not change in all these solutions after standing for 7 days, revealing the good stability of SeCQDs.

### The ROS-Scavenging Activity of SeCQDs

**FIGURE 3:**
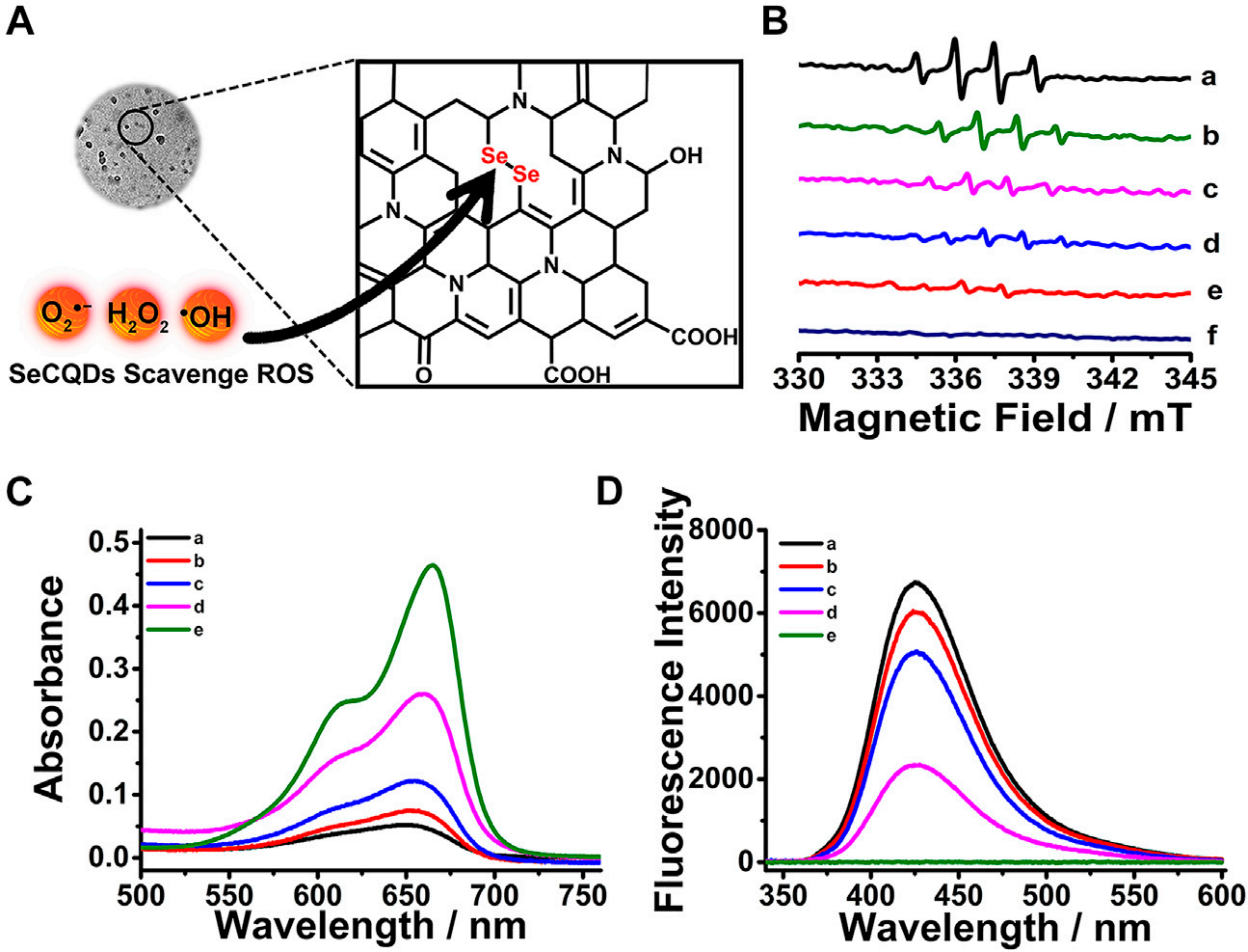
Scheme showing the ROS-scavenging activity of SeCQDs **(A)**. ESR spectra of DMPO/•OH adducts were collected from different samples **(B)**. a, H_2_O_2_-UV; b, H_2_O_2_ + UV + 5 μg ml^−1^ SeCQDs; c, H_2_O_2_ + UV + 10 μg ml^−1^ SeCQDs; d, H_2_O_2_ + UV + 50 μg ml^−1^ SeCQDs; e, UV + 50 μg ml^−1^ SeCQDs; f, 50 μg ml^−1^ SeCQDs. The formation of •OH monitored by MB assay **(C)** and TA assay **(D)** under different conditions. a, Probe + H_2_O_2_ + UV; b, Probe + H_2_O_2_ + UV + 5 μg ml^−1^ SeCQDs; c, Probe + H_2_O_2_ + UV + 10 μg ml^−1^ SeCQDs; d, Probe + H_2_O_2_ + UV + 50 μg ml^−1^ SeCQDs; e, Probe.

Following the synthesis and analysis of SeCQDs, we next evaluated their ROS-scavenging activity (Figure 3A). Among all the ROS, ⋅OH as one of the most damaging species, can directly react with almost all biomolecules including DNA, proteins and membrane lipids (Li et al., 2017). Thus, we employed ⋅OH as a model to investigate the radical scavenging efficiency of SeCQDs using ESR spectroscopy and colorimetric as well as fluorescent assay. As shown in Figure 3B, in the presence of the spin trap, DMPO, a strong typical signature of DMPO-HO⋅ appeared in the system of H_2_O_2_ with UV irradiation. However, the amount of produced ⋅OH radical decreased significantly upon addition of SeCQDs. And the inhibition effect on the formation of ⋅OH radical was highly dependent on the concentration of SeCQDs. The same results were obtained from the colorimetric and fluorescent assay, in which MB (Zhu et al., 2020) and TA (Karim et al., 2018) were utilized as the dyes, respectively (Figures 3C,D). The discoloration of MB and the decrease in fluorescence intensity of TAOH produced via the oxidation of TA by ⋅OH both indicated the ROS-scavenging activity of SeCQDs.

### Effect of SeCQDs on Aβ Aggregation

**FIGURE 4:**
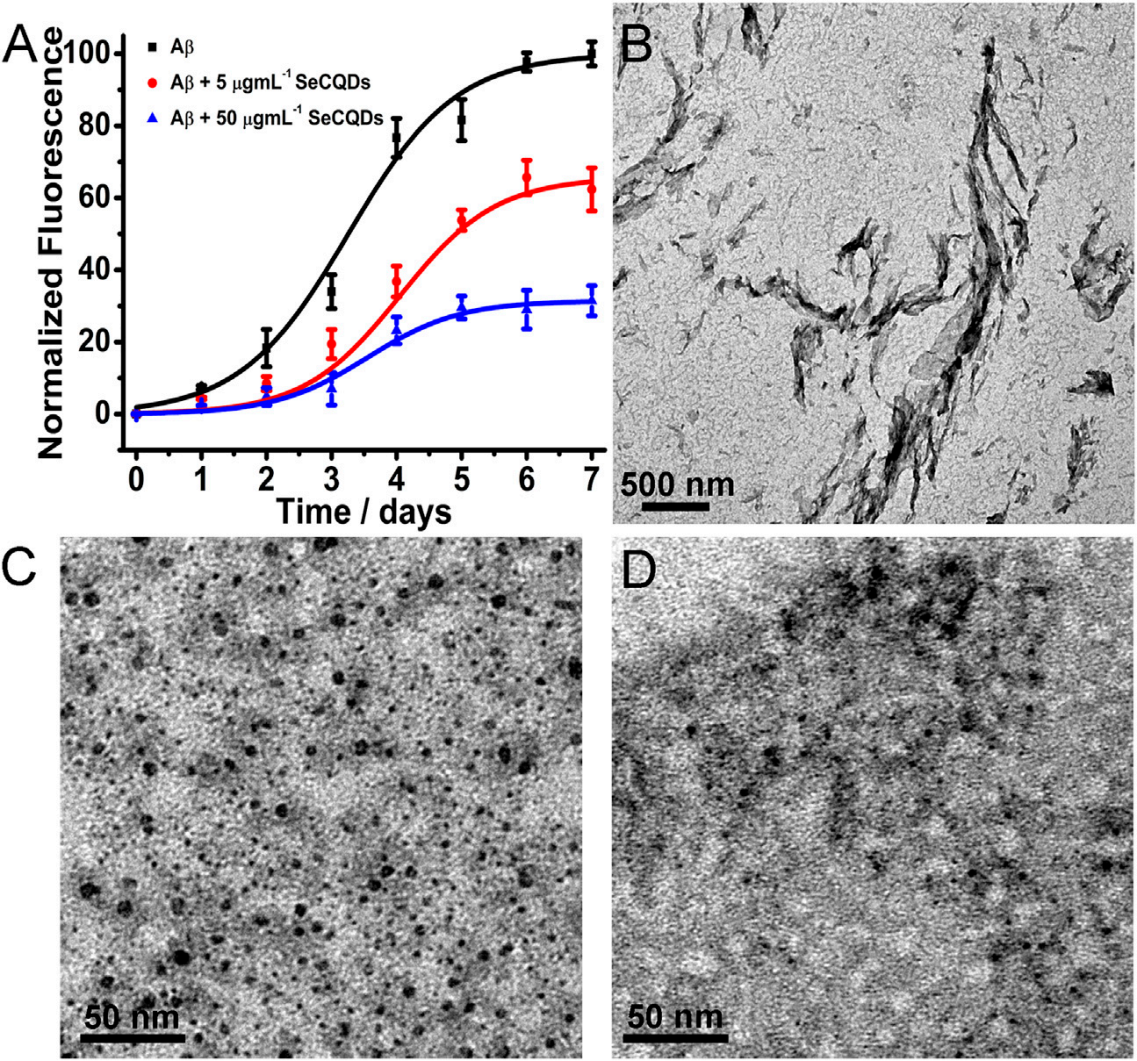
Fibrillation kinetics of Aβ40 in the absence or presence of SeCQDs monitored by ThT **(A)**. The Aβ40 concentration was 100 μM. The morphology of Aβ40 aggregates was analyzed by TEM images: 100 μM Aβ40 **(B)**, 100 μM Aβ40 in the presence of 5 μg ml^−1^ SeCQDs **(C)**, 100 μM Aβ40 in the presence of 50 μg ml^−1^ SeCQDs **(D)**.

ThT fluorescence assay was employed to examine the influence of SeCQDs on the aggregation of Aβ. Aβ40, the most abundantly produced Aβ isoform, was chosen as the protein model, which has been widely used for *in vitro* amyloidogenesis study (Gao et al., 2019; Li et al., 2020). As a benzothiazole dye, ThT can selectively bind to the β-sheet region present in Aβ fibrils, resulting in a strong increase in its fluorescence. The ThT fluorescence curves in Figure 4A clearly indicated the inhibition effect of SeCQDs on Aβ aggregation. When fresh Aβ40 alone incubated at 37°C, the ThT fluorescence displayed a standard sigmoidal curve, consistent with the nucleation-dependent polymerization model (Gao et al., 2019). However, the increasing trend of ThT fluorescence was greatly suppressed upon introduction of SeCQDs, which indicated that the aggregation process of Aβ was inhibited by SeCQDs. A control experiment was also carried out to clarify that the fluorescence of ThT could not be affected by the addition of SeCQDs with the concentration used in the inhibition study (Supplementary Figure S5). In addition, SeCQDs suppressed Aβ aggregation in a dose-dependent manner (Figure 4A). Critically, compared with SeCQDs, SeCys displayed little inhibition effect on Aβ aggregation (Supplementary Figure S6). The inhibition on Aβ40 aggregation was further evaluated by circular dichroism (CD) spectra. As demonstrated in Supplementary Figure S7, SeCQDs could inhibit structural transition from the native Aβ40 random coil to the β-sheet conformation in solution. While, Aβ40 retained monomeric forms at the start stage of the experiment and SeCQDs themselves did not show any obvious signal in this range, which eliminated the influence of Aβ40 and SeCQDs themselves on the inhibition results (Supplementary Figure S8). TEM analysis also showed that in the absence of SeCQDs, Aβ monomers assembled into mature fibrils, while, SeCQDs predominantly inhibited Aβ fibrillization (Figures 4B–D).

### The Binding Models Between SeCQDs and Aβ

**FIGURE 5:**
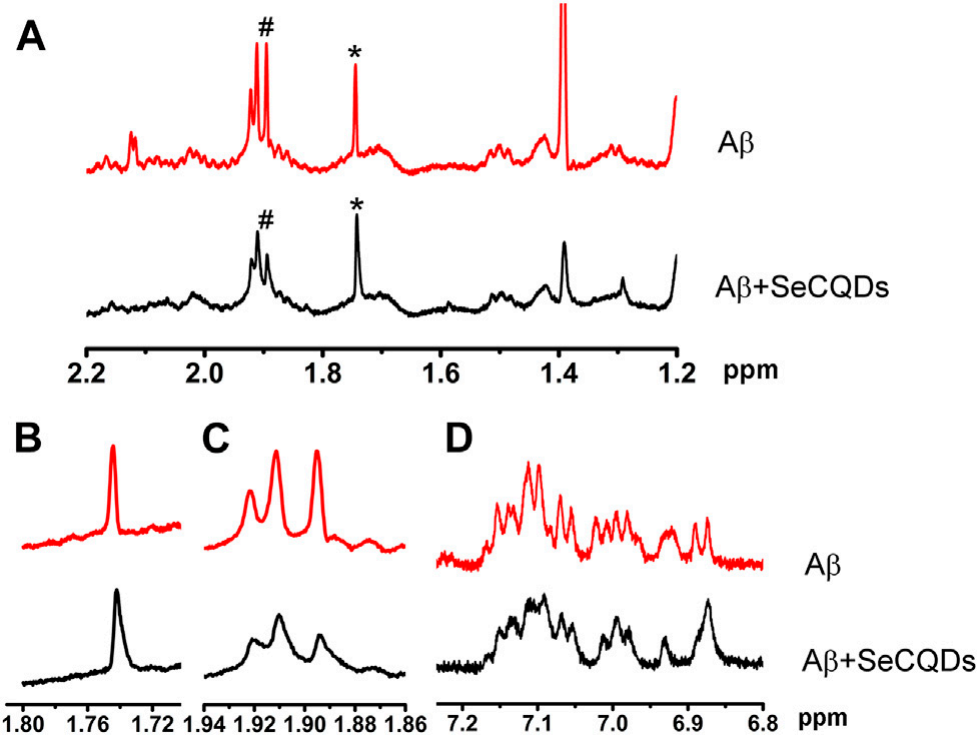
^1^H NMR spectra of Aβ before and after incubated with SeCQDs. The signals of amide protons of Lys16 (the peaks marked with *) and the resonances from the protons of Glu (the peaks marked with #) were restored upon co-incubation of SeCQDs with Aβ **(A)**. Locally amplified ^1^H NMR spectra centered at 1.74 ppm **(B)**. Locally amplified ^1^H NMR spectra centered at 1.91 ppm **(C)**. The changes of the resonances from the protons of Tyr10, Phe19 and Phe20 **(D)**.

The inhibition behavior and binding models between SeCQDs and Aβ were further confirmed by NMR spectroscopy (Zagorski and Barrow, 1992; Amir et al., 2012; Li et al., 2014; Valiente-Gabioud et al., 2018). Compared with that of Aβ alone, the ^1^H NMR signals of all His proton lines and Tyr10 lines in Aβ were broadened and the resonances of Lys and Glu were shifted to high-field upon treated with SeCQDs (Figure 5). The changes in chemical shift indicated an altered chemical environment due to the direct interaction with SeCQDs or due to a structural change of Aβ that occurred upon binding. The negative charged SeCQDs (Supplementary Figure S9) can easily bind to the cationic cluster HHQK of Aβ via electrostatic interactions. In addition, the paired α-carboxyl and amino groups existed on the edge of SeCQDs camouflaged them as large amino acids, which can also trigger multivalent interactions with these amino acids. Due to the charged nature and bulky size, SeCQDs can cause the structure of Aβ to be changed, endowing them with inhibition effects on Aβ aggregation.

### The Rescuing Effects of SeCQDs on Aβ-Induced Cytotoxicity

**FIGURE 6:**
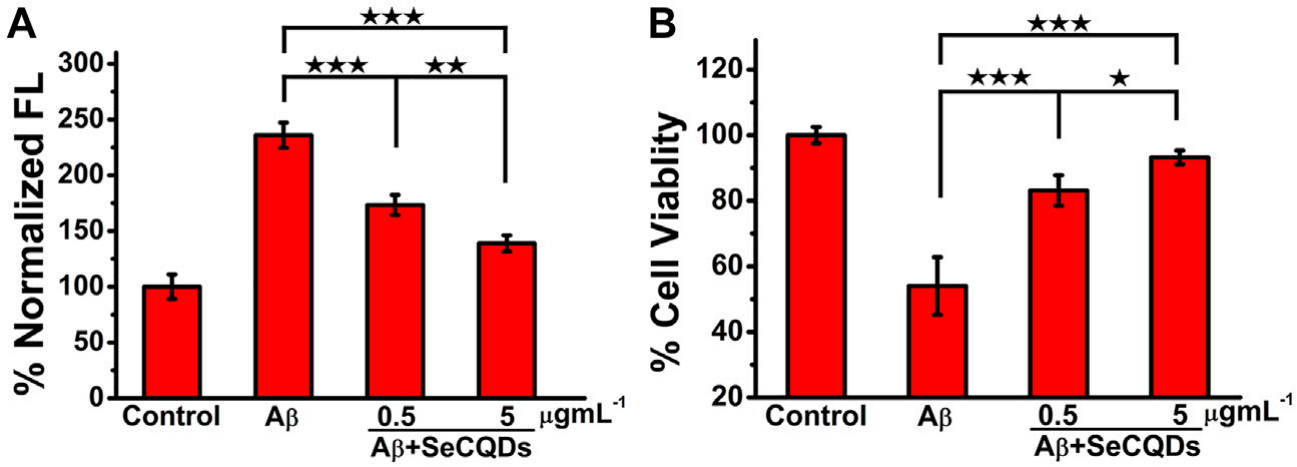
Effect of the SeCQDs on intracellular ROS formation in Aβ40-treated PC12 cells **(A)**. Protection effects of SeCQDs on Aβ40-induced cytotoxicity of PC12 cells **(B)**. The concentration of Aβ40 was 10 μM. The control group was Aβ40 untreated cells. Data represents mean ± SEM. *p < 0.05, **p < 0.01, ***p < 0.001.

Having demonstrated the ability of SeCQDs to inhibit the formation of ROS and Aβ aggregation, we next investigated whether they could reduce the production of ROS in cells caused by Aβ aggregates as well as the cytotoxicity of Aβ aggregates. As shown in Figure 6A, the content of ROS in cells treated with Aβ aggregates increased significantly up to 236%, relative to that in the untreated control cells. However, upon pretreatment with SeCQDs at the concentration of 0.5 μg ml^−1^ or 5 μg ml^−1^, the intracellular level of ROS largely decreased to 173 and 139%, respectively, indicating SeCQDs can greatly reduce the intracellular production of ROS in a dose-dependent manner. Critically, there was no significant difference between the generation of ROS in SeCQDs solely treated cells and the untreated cells (Supplementary Figure S10). The effect of SeCQDs on the cytotoxicity of Aβ aggregates was also investigated. Figure 6B indicated that compared to the untreated cells, the cell viability was reduced to 54% upon treatment with Aβ aggregates for 24 h. SeCQDs protected PC12 cells from Aβ aggregates-induced cell death in a concentration-dependent manner. Importantly, the cytotoxicity of SeCQDs was also examined in PC12 cells. As shown in Supplementary Figure S11, SeCQDs showed no toxicity in our experimental conditions, suggesting that SeCQDs can be utilized as potential agents for AD treatment. It is well known that Aβ aggregates can cause damage to mitochondrial structure and function, which is one of the neurotoxic mechanisms of Aβ (Godoy et al., 2017). Mitochondrial membrane potential (MMP) was used to evaluate the function of mitochondria via the fluorescent probe, JC-1 (Liu et al., 2017; Shen et al., 2020). Supplementary Figure S12 showed that SeCQDs can significantly alleviate the Aβ induced depolarization of MMP. All these results proved the neuroprotective effect of SeCQDs on PC12 cells.

### *In Vivo* Biodistribution and Biocompatibility

**FIGURE 7:**
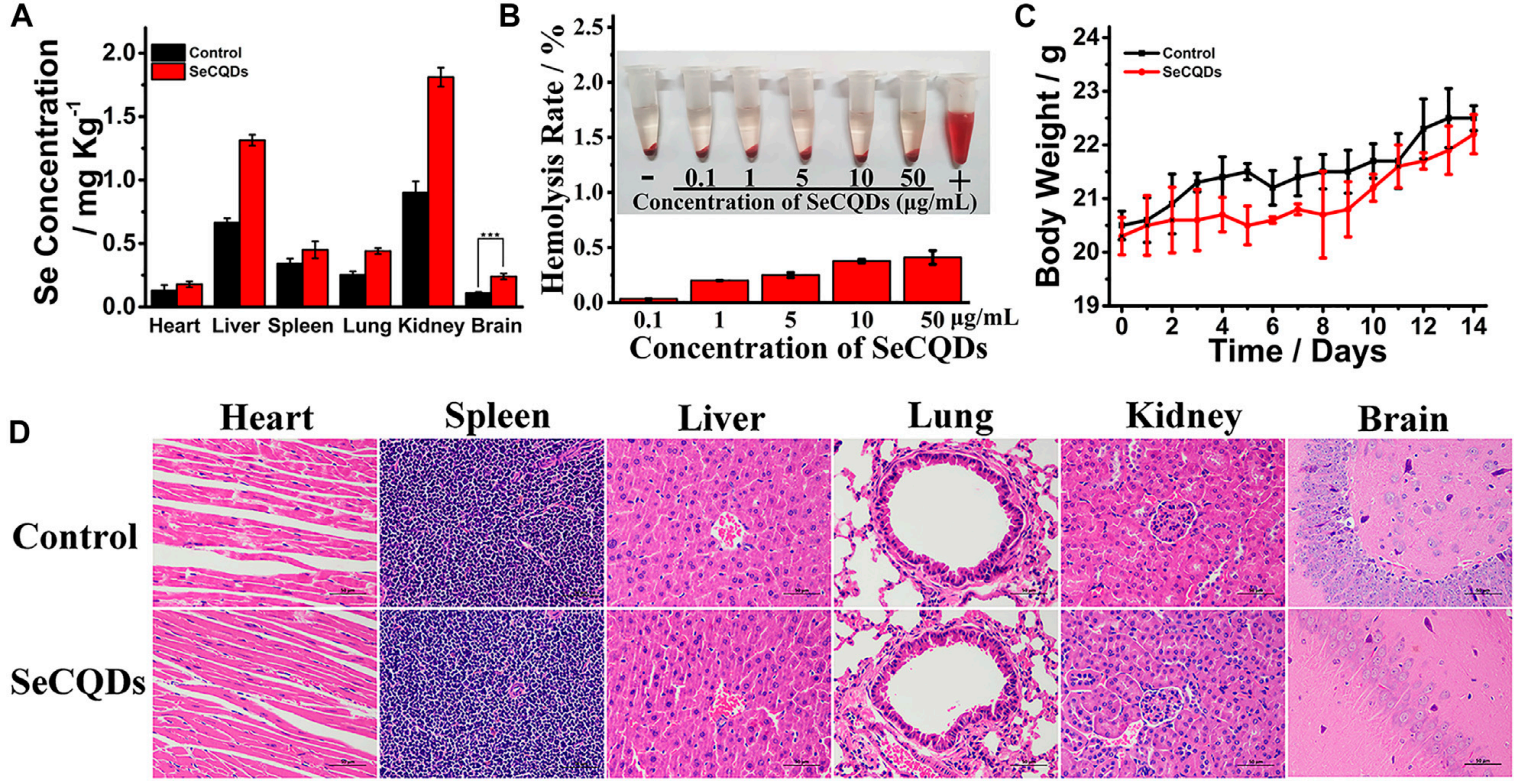
The biodistribution and biocompatibility of SeCQDs. The biodistribution of SeCQDs in healthy mice at 6 h after i. v. injection based on ICP-MS analysis **(A)**. Hemolytic assays for SeCQDs. The RBCs were collected by centrifugation of heparin-stabilized mice blood samples **(B)**. The concentration of SeCQDs varied from 0.1 μg ml^−1^–50 μg ml^−1^. PBS and water which incubated with the diluted RBC suspension were served as negative and positive controls, respectively. Relative body weight of healthy mice after the treatment of SeCQDs in 14 days **(C)** (Mean ± SD, n = 3). *In vivo* long-term toxicology of SeCQDs. H&E images of major organs obtained from the mice after intravenous injection with SeCQDs at 14 days post-injection **(D)**. Scale bars are 50 μm.

To ensure the possibility and safety for AD treatment, it is of importance to study the *in vivo* biodistribution and biocompatibility of SeCQDs. To investigate the accurate distribution of SeCQDs in main organs, SeCQDs were intravenously injected into C57BL/6 mice, and the main organs were collected after 6 h injection to determine the remained Se by ICP-MS (Figure 7A). The accumulation of SeCQDs in the brain revealed the potential capability of SeCQDs to cross the BBB.

Hemolysis testing has been recognized as a classical assay for evaluating the cell damage effect of nanomaterials (Zhu et al., 2019; Zhou et al., 2020). As shown in Figure 7B, SeCQDs with the concentration varied from 0.1 μg ml^−1^–50 μg ml^−1^ only caused <1% haemolysis, which was considered to be biocompatible in accordance with ISO/TR 7406 (the permissible limit for hemolysis is 5%) (Chibhabha et al., 2020; Lima et al., 2021). The *in vivo* biocompatibility of SeCQDs was also analyzed by the change of body weight after injecting the drug (Figure 7C). No significant difference in body weight was observed between SeCQDs-treated mice and untreated mice within 14 days. On day 14, the main organs of the mice were collected and examined by Hematoxylin-eosin (H&E) staining (Figure 7D). H&E stained pathological sections of the main organs of SeCQDs treated mice including heart, liver, spleen, lung, kidney and brain exhibited no apparent lesions or abnormalities as compared with that from the untreated group, which demonstrated the excellent *in vivo* biocompatibility of SeCQDs.

### The SeCQDs Improve Cognitive Ability in AD Model Rats

**FIGURE 8:**
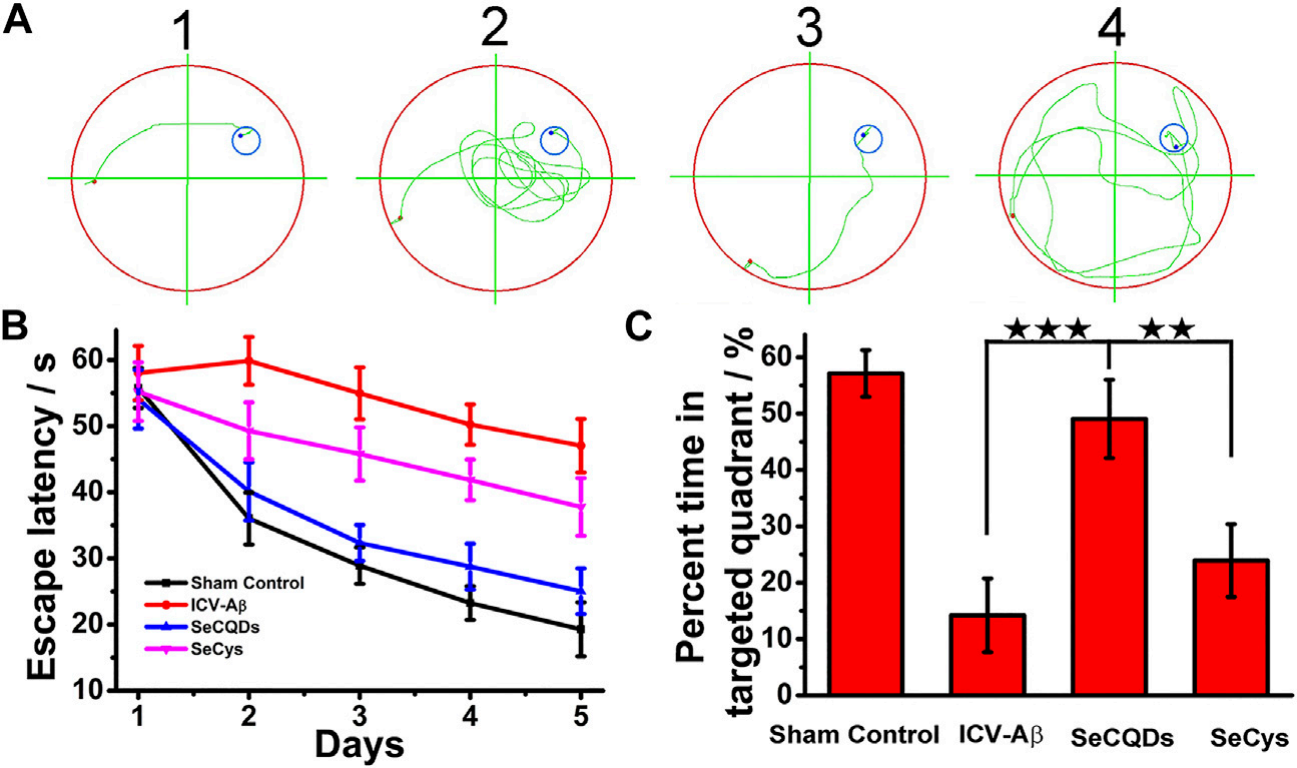
The effect of SeCQDs on the cognitive functions of the rats induced by Aβ via the Morris water maze test. The swimming trace to find the hidden platform **(A)**. The escape latency to find the hidden platform during the 5-days training **(B)**. The percent time spent in the target quadrant after 5 days training **(C)**. Data represents mean ± SEM. *p < 0.05, **p < 0.01, ***p < 0.001.

Inspired by the excellent biocompatibility of SeCQDs and their *in vitro* inhibition effect on Aβ-induced cytotoxicity, we next investigated whether SeCQDs can be used for highly efficient *in vivo* AD treatment. Progressive cognitive declines, the main clinical symptoms of AD, have been reported to be directly correlated with Aβ-mediated synaptic deficits (Beckman et al., 2019; Hou et al., 2020; Samanta et al., 2021). In order to analyze the possibility of SeCQDs for *in vivo* AD therapy, the Morris water maze (MWM) test (Sanati et al., 2019; Sun et al., 2019; Huang et al., 2021) was first conducted to evaluate the efficiency of SeCQDs to ameliorate Aβ induced memory deficits. As shown in Figure 8A, B, all the rats exhibited progressive decline in escape latencies during the 5-days spatial learning training. Compared with the normal saline injected wild type rats (control group), Aβ-infused rats required much more time to find the hidden platform (Figure 8B) and spent decreased time in the target platform quadrant (Figure 8C) in the following the probe trial test, indicating that Aβ induced spatial learning and memory deficits in rats. In contrast, after administrated with SeCQDs, the Aβ40-infused rats showed shorter escape latencies and increased staying time in the targeted quadrant. Critically, compared with SeCys, SeCQDs showed obviously higher efficiency in improving the cognitive ability of Aβ40-infused rats.

**FIGURE 9:**
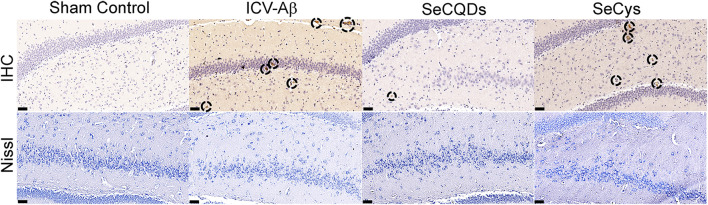
The IHC analysis of Aβ deposition (upper) and the Nissl staining of nerve cells (down) in the brains of sham control rat, Aβ40-infused rat, Aβ40-infused rat treated with SeCQDs and SeCys. Scale bars are 50 μm.

Aβ deposition and neuronal loss in the hippocampus have been widely known as the key markers of AD. Aβ deposition in the brain was characterized by immunohistochemistry (IHC) of Aβ (Sun et al., 2019; Liu et al., 2019). As displayed in Figure 9, obvious Aβ deposition was observed around the neurons in Aβ-infused rats. However, Aβ plaques depositions were considerably decreased in SeCQDs-treated groups, indicating that administration of SeCQDs reduced Aβ accumulation in the brain. Nissl staining of hippocampal slices (Sun et al., 2019; Liu et al., 2019) showed that Aβ-infused rats had very few Nissl bodies. However, more neurons with restored integrity were observed in SeCQDs-treated AD model rats, which confirmed the protective effect of SeCQDs against Aβ induced neurodegenerative consequences (Figure 9). Critically, the protection effects were also observed in SeCys-treated groups but not as obvious as that of SeCQDs-treated group. Similar to other selenium compounds, short-term administration of SeCys also showed the potential in improving memory deficits and reducing Aβ plaques in AD model mice (Weekley and Harris, 2013; Du et al., 2016). However, the relative high toxicity limited its clinical application as therapeutic drug for AD treatment (Supplementary Figure S13). In contrast, with the intrinsic properties of both selenium and CQDs, SeCQDs possessed excellent biocompatibility and can significantly improve the reference memory deficit, inhibit Aβ accumulation and neuron degeneration in Aβ-treated rats. All these characteristics rendered SeCQDs as promising candidates for AD therapy.

## Conclusion

In summary, with the anti-aggregation property and antioxidant effect, the large amino acid mimicking SeCQDs were employed as novel agents for AD treatment. The SeCQDs displayed good biocompatibility and a remarkable ROS-scavenging activity. Moreover, the remained α-carboxyl and amino groups on edge of SeCQDs triggered multivalent interactions with Aβ, leading to the ability of SeCQDs to inhibit Aβ aggregation. *In vivo* study demonstrated that SeCQDs can also ameliorate the Aβ induced memory deficits, reduce Aβ accumulation and inhibit neuron degeneration in AD model rats. This finding may open a new avenue for the design of multifunctional nanoagents for AD therapy. Furthermore, with the potential ability to cross the BBB and minimal toxicity, SeCQDs have potential for translation into clinical applications for treatment of various central nervous system diseases not only AD.

## Data Availability

The original contributions presented in the study are included in the article/Supplementary Material, further inquiries can be directed to the corresponding author.

## References

[B1] Amir A. Shmuel E. Zagalsky R. Sayer A. H. Nadel Y. Fischer B. (2012). Nucleoside-5'-phosphorothioate Analogues Are Biocompatible Antioxidants Dissolving Efficiently Amyloid Beta-Metal Ion Aggregates. Dalton Trans. 41, 8539–8549. 10.1039/C2DT30631J 22652964

[B2] Ashrafizadeh M. Mohammadinejad R. Kailasa S. K. Ahmadi Z. Afshar E. G. Pardakhty A. (2020). Carbon Dots as Versatile Nanoarchitectures for the Treatment of Neurological Disorders and Their Theranostic Applications: a Review. Adv. Colloid Interf. Sci. 278, 102123. 10.1016/j.cis.2020.102123 32087367

[B3] Beckman D. Ott S. Donis-Cox K. Janssen W. G. Bliss-Moreau E. Rudebeck P. H. (2019). Oligomeric Aβ in the Monkey Brain Impacts Synaptic Integrity and Induces Accelerated Cortical Aging. Proc. Natl. Acad. Sci. U S A. 116, 26239–26246. 10.1073/pnas.1902301116 PMC6936351 31871145

[B4] Chibhabha F. Yang Y. Ying K. Jia F. Zhang Q. Ullah S. (2020). Non-invasive Optical Imaging of Retinal Aβ Plaques Using Curcumin Loaded Polymeric Micelles in APPswe/PS1ΔE9 Transgenic Mice for the Diagnosis of Alzheimer's Disease. J. Mater. Chem. B 8, 7438–7452. 10.1039/D0TB01101K 32662804

[B5] Devi P. Saini S. Kim K. H. (2019). The Advanced Role of Carbon Quantum Dots in Nanomedical Applications. Biosens. Bioelectron. 141, 111158. 10.1016/j.bios.2019.02.059 31323605

[B6] Du X. Wang C. Liu Q. (2016). Potential Roles of Selenium and Selenoproteins in the Prevention of Alzheimer's Disease. Curr. Top. Med. Chem. 16, 835–848. 10.2174/1568026615666150827094936 26311427

[B7] Du Z. Li M. Ren J. Qu X. (2021). Current Strategies for Modulating Aβ Aggregation with Multifunctional Agents. Acc. Chem. Res. 54, 2172–2184. 10.1021/acs.accounts.1c00055 33881820

[B8] Gao N. Du Z. Guan Y. Dong K. Ren J. Qu X. (2019). Chirality-selected Chemical Modulation of Amyloid Aggregation. J. Am. Chem. Soc. 141, 6915–6921. 10.1021/jacs.8b12537 30969760

[B9] Godoy J. A. Lindsay C. B. Quintanilla R. A. Carvajal F. J. Cerpa W. Inestrosa N. C. (2017). Quercetin Exerts Differential Neuroprotective Effects against H2O2 and Aβ Aggregates in Hippocampal Neurons: the Role of Mitochondria. Mol. Neurobiol. 54, 7116–7128. 10.1007/s12035-016-0203-x 27796749

[B10] Hou K. Zhao J. Wang H. Li B. Li K. Shi X. (2020). Chiral Gold Nanoparticles Enantioselectively rescue Memory Deficits in a Mouse Model of Alzheimer's Disease. Nat. Commun. 11, 4790. 10.1038/s41467-020-18525-2 32963242 PMC7509831

[B11] Huang D. Cao Y. Yang X. Liu Y. Zhang Y. Li C. (2021). A Nanoformulation-Mediated Multifunctional Stem Cell Therapy with Improved Beta-Amyloid Clearance and Neural Regeneration for Alzheimer's Disease. Adv. Mater. 33, e2006357. 10.1002/adma.202006357 33624894

[B12] Iraji A. Khoshneviszadeh M. Firuzi O. Khoshneviszadeh M. Edraki N. (2020). Novel Small Molecule Therapeutic Agents for Alzheimer Disease: Focusing on BACE1 and Multi-Target Directed Ligands. Bioorg. Chem. 97, 103649. 10.1016/j.bioorg.2020.103649 32101780

[B13] Jokar S. Khazaei S. Behnammanesh H. Shamloo A. Erfani M. Beiki D. (2019). Recent Advances in the Design and Applications of Amyloid-β Peptide Aggregation Inhibitors for Alzheimer's Disease Therapy. Biophys. Rev. 11, 901–925. 10.1007/s12551-019-00606-2 31713720

[B14] Karim M. N. Anderson S. R. Singh S. Ramanathan R. Bansal V. (2018). Nanostructured Silver Fabric as a Free-Standing NanoZyme for Colorimetric Detection of Glucose in Urine. Biosens. Bioelectron. 110, 8–15. 10.1016/j.bios.2018.03.025 29574249

[B15] Kim K. Kim M. J. Kim D. W. Kim S. Y. Park S. Park C. B. (2020). Clinically Accurate Diagnosis of Alzheimer's Disease via Multiplexed Sensing of Core Biomarkers in Human Plasma. Nat. Commun. 11, 119. 10.1038/s41467-019-13901-z 31913282 PMC6949261

[B16] Lei L. Zou Z. Liu J. Xu Z. Fu Y. Tian Y. (2021). Multifunctional Peptide-Assembled Micelles for Simultaneously Reducing Amyloid-β and Reactive Oxygen Species. Chem. Sci. 12, 6449–6457. 10.1038/s41593-019-0372-910.1039/d1sc00153a 34084446 PMC8115327

[B17] Li F. Li T. Sun C. Xia J. Jiao Y. Xu H. (2017). Selenium-doped Carbon Quantum Dots for Free-Radical Scavenging. Angew. Chem. Int. Ed. Engl. 56, 9910–9914. 10.1002/anie.201705989 28643462

[B18] Li M. Howson S. E. Dong K. Gao N. Ren J. Scott P. (2014). Chiral Metallohelical Complexes Enantioselectively Target Amyloid β for Treating Alzheimer's Disease. J. Am. Chem. Soc. 136, 11655–11663. 10.1021/ja502789e 25062433

[B19] Li M. Liu Z. Ren J. Qu X. (2020). Molecular Crowding Effects on the Biochemical Properties of Amyloid β-heme, Aβ-Cu and Aβ-Heme-Cu Complexes. Chem. Sci. 11, 7479–7486. 10.1039/D0SC01020K 34123030 PMC8159413

[B20] Lima A. C. Campos C. F. Cunha C. Carvalho A. Reis R. L. Ferreira H. (2021). Biofunctionalized Liposomes to Monitor Rheumatoid Arthritis Regression Stimulated by Interleukin-23 Neutralization. Adv. Healthc. Mater. 10, e2001570. 10.1002/adhm.202001570 33103383

[B21] Liu J. Jin C. Yuan B. Chen Y. Liu X. Ji L. (2017). Enhanced Cancer Therapy by the Marriage of Metabolic Alteration and Mitochondrial-Targeted Photodynamic Therapy Using Cyclometalated Ir(iii) Complexes. Chem. Commun. (Camb) 53, 9878–9881. 10.1039/C7CC05518H 28825071

[B22] Liu R. Yang J. Liu L. Lu Z. Shi Z. Ji W. (2019). An "Amyloid-β Cleaner" for the Treatment of Alzheimer's Disease by Normalizing Microglial Dysfunction. Adv. Sci. (Weinh) 7, 1901555. 10.1002/advs.201901555 31993283 PMC6974948

[B23] Liu T. Xu L. He L. Zhao J. Zhang Z. Chen Q. (2020). Selenium Nanoparticles Regulates Selenoprotein to Boost Cytokine-Induced Killer Cells-Based Cancer Immunotherapy. Nano Today 35, 100975. 10.1016/j.nantod.2020.100975

[B24] Mahmoud N. N. Albasha A. Hikmat S. Hamadneh L. Zaza R. Shraideh Z. (2020). Nanoparticle Size and Chemical Modification Play a Crucial Role in the Interaction of Nano Gold with the Brain: Extent of Accumulation and Toxicity. Biomater. Sci. 8, 1669–1682. 10.1039/C9BM02072A 31984985

[B25] Menon S. Devi Ks S. Santhiya R. Rajeshkumar S. Venkat Kumar S. (2018). Selenium Nanoparticles: a Potent Chemotherapeutic Agent and an Elucidation of its Mechanism. Colloids Surf. B Biointerfaces 170, 280–292. 10.1016/j.colsurfb.2018.06.006 29936381

[B26] Ono K. Hamaguchi T. Naiki H. Yamada M. (2006). Anti-amyloidogenic Effects of Antioxidants: Implications for the Prevention and Therapeutics of Alzheimer's Disease. Biochim. Biophys. Acta 1762, 575–586. 10.1016/j.bbadis.2006.03.002 16644188

[B27] Palop J. J. Mucke L. (2010). Amyloid-beta-induced Neuronal Dysfunction in Alzheimer's Disease: from Synapses toward Neural Networks. Nat. Neurosci. 13, 812–818. 10.1038/nn.2583 20581818 PMC3072750

[B28] Rao S. Lin Y. Du Y. He L. Huang G. Chen B. (2019). Designing Multifunctionalized Selenium Nanoparticles to Reverse Oxidative Stress-Induced Spinal Cord Injury by Attenuating ROS Overproduction and Mitochondria Dysfunction. J. Mater. Chem. B 7, 2648–2656. 10.1039/c8tb02520g 32254998

[B29] Rosenkrans Z. T. Sun T. Jiang D. Chen W. Barnhart T. E. Zhang Z. (2020). Selenium-doped Carbon Quantum Dots Act as Broad-Spectrum Antioxidants for Acute Kidney Injury Management. Adv. Sci. (Weinh) 7, 2000420. 10.1002/advs.202000420 32596126 PMC7312409

[B30] Samanta S. Rajasekhar K. Ramesh M. Murugan N. A. Alam S. Shah D. (2021). Naphthalene Monoimide Derivative Ameliorates Amyloid burden and Cognitive Decline in a Transgenic Mouse Model of Alzheimer's Disease. Adv. Therap. 4, 2000225. 10.1002/adtp.202000225

[B31] Sanati M. Khodagholi F. Aminyavari S. Ghasemi F. Gholami M. Kebriaeezadeh A. (2019). Impact of Gold Nanoparticles on Amyloid β-Induced Alzheimer's Disease in a Rat Animal Model: Involvement of STIM Proteins. ACS Chem. Neurosci. 10, 2299–2309. 10.1021/acschemneuro.8b00622 30933476

[B32] Shen J. Rees T. W. Zhou Z. Yang S. Ji L. Chao H. (2020). A Mitochondria-Targeting Magnetothermogenic Nanozyme for Magnet-Induced Synergistic Cancer Therapy. Biomaterials 251, 120079. 10.1016/j.biomaterials.2020.120079 32387686

[B33] Sun J. Wei C. Liu Y. Xie W. Xu M. Zhou H. (2019). Progressive Release of Mesoporous Nano-Selenium Delivery System for the Multi-Channel Synergistic Treatment of Alzheimer's Disease. Biomaterials 197, 417–431. 10.1016/j.biomaterials.2018.12.027 30638753

[B34] Tönnies E. Trushina E. (2017). Oxidative Stress, Synaptic Dysfunction, and Alzheimer's Disease. J. Alzheimers Dis. 57, 1105–1121. 10.3233/JAD-161088 28059794 PMC5409043

[B35] Valiente-Gabioud A. A. Riedel D. Outeiro T. F. Menacho-Márquez M. A. Griesinger C. Fernández C. O. (2018). Binding Modes of Phthalocyanines to Amyloid β Peptide and Their Effects on Amyloid Fibril Formation. Biophys. J. 114, 1036–1045. 10.1016/j.bpj.2018.01.003 29539391 PMC5883547

[B36] Weekley C. M. Harris H. H. (2013). Which Form Is that? the Importance of Selenium Speciation and Metabolism in the Prevention and Treatment of Disease. Chem. Soc. Rev. 42, 8870–8894. 10.1039/C3CS60272A 24030774

[B37] Yu B. Li H. Zhang J. Zheng W. Chen T. (2015). Rational Design and Fabrication of a Cancer-Targeted Chitosan Nanocarrier to Enhance Selective Cellular Uptake and Anticancer Efficacy of Selenocystine. J. Mater. Chem. B 3, 2497–2504. 10.1039/C4TB02146K 32262124

[B38] Zagorski M. G. Barrow C. J. (1992). NMR Studies of Amyloid .beta.-peptides: Proton Assignments, Secondary Structure, and Mechanism of an .alpha.-helix Fwdarw. beta.-sheet Conversion for a Homologous, 28-residue, N-Terminal Fragment. Biochemistry 31, 5621–5631. 10.1021/bi00139a028 1610809

[B39] Zeng H. Qi Y. Zhang Z. Liu C. Peng W. Zhang Y. (2021). Nanomaterials toward the Treatment of Alzheimer's Disease: Recent Advances and Future Trends. Chin. Chem. Lett. 32, 1857–1868. 10.1016/j.cclet.2021.01.014

[B40] Zhang J. Zhou X. Yu Q. Yang L. Sun D. Zhou Y. (2014). Epigallocatechin-3-gallate (EGCG)-stabilized Selenium Nanoparticles Coated with Tet-1 Peptide to Reduce Amyloid-β Aggregation and Cytotoxicity. ACS Appl. Mater. Inter. 6, 8475–8487. 10.1021/am501341u 24758520

[B41] Zhang P. Kishimoto Y. Grammatikakis I. Gottimukkala K. Cutler R. G. Zhang S. (2019). Senolytic Therapy Alleviates Aβ-Associated Oligodendrocyte Progenitor Cell Senescence and Cognitive Deficits in an Alzheimer's Disease Model. Nat. Neurosci. 22, 719–728. 10.1038/s41593-019-0372-9 30936558 PMC6605052

[B42] Zhou H. Gong Y. Liu Y. Huang A. Zhu X. Liu J. (2020). Intelligently Thermoresponsive Flower-like Hollow Nano-Ruthenium System for Sustained Release of Nerve Growth Factor to Inhibit Hyperphosphorylation of Tau and Neuronal Damage for the Treatment of Alzheimer's Disease. Biomaterials 237, 119822. 10.1016/j.biomaterials.2020.119822 32035322

[B43] Zhu W. Guo J. Agola J. O. Croissant J. G. Wang Z. Shang J. (2019). Metal-organic Framework Nanoparticle-Assisted Cryopreservation of Red Blood Cells. J. Am. Chem. Soc. 141, 7789–7796. 10.1021/jacs.9b00992 31017405

[B44] Zhu X. Liu Y. Yuan G. Guo X. Cen J. Gong Y. (2020). *In Situ* fabrication of MS@MnO2 Hybrid as Nanozymes for Enhancing ROS-Mediated Breast Cancer Therapy. Nanoscale 12, 22317–22329. 10.1039/D0NR03931D 33146638

