# Supplementary material for: Large Amino Acid Mimicking Selenium-Doped Carbon Quantum Dots for Multi-Target Therapy of Alzheimer’s Disease
Source: Front Pharmacol. 2021 Oct 27;12:778613. doi: 10.3389/fphar.2021.778613 (PMC8579002; doi:10.3389/fphar.2021.778613)
Supplement: Supplementary file 1 [file DataSheet1.docx]

Supplementary Material

**Reagents and Materials**

Aβ40 was purchased from Enzo Life Sciences. L-Selenocystine (SeCys) and Thioflavin T (ThT) were bought from J&K Chemicals. 1,1,1,3,3,3-Hexafluoro-2-propanol (HFIP) was obtained from Acros organics. Methylene blue (MB), disodium terephthalate (TA) and 5,5-dimethyl-1-pyrroline-N-oxide (DMPO) were obtained from Aladdin (Shanghai, China). H_2_O_2_ was obtained from Laiyang Kangde Chemical Co., Ltd.. Sodium pentobarbital was purchased from Siegfried. All reagents and solvents were used as received from commercial suppliers without further purification. The solutions used in this study were prepared with deionized water (D.I. water, 18.2 MΩ cm) from a Milli-Q system (Millipore, Bedford, MA).

**Measurements**

The UV-Vis absorption spectra were monitored by a Shimadzu UV-2700 spectrophotometer (Japan). Fluorescence spectra were recorded on a fluorescence spectrophotometer (F-380, Gangdong Technology, China). The X-ray diffraction (XRD) measurement was recorded using a SmartLab Focus diffractometer (Rigaku) with Cu Kα source (λ = 1.541862 Å). Transmission Electron Microscope (TEM) images were recorded using a Hitachi 7650B microscope operating at 200 kV. X-ray photoelectron spectroscopy (XPS) analysis was carried on ESCALAB 250xi photoelectron spectrometer equipped with an Al Kα monochromated X-ray source (Thermo Scientific, USA). Fourier translation infrared (FT-IR) spectra were obtained on a SHIMADZU FT-IR-8400S. Electron paramagnetic resonance (EPR) spectra were measured via a magnettech MS-5000 EPR spectrometer (Germany). Circular dichroism (CD) spectra were monitored by a J-1500 CD spectrophotometer (JASCO) with the scanning speed of 10 nm min^-1^ and data pitch of 0.5 nm.

**Peptide Preparation**

Aβ40 (lot no. ALX-151-003) was obtained from Enzo Life Sciences and prepared according to previous reports (Palop and Mucke, 2010; Kim et al., 2020). Briefly, the peptide was dissolved in HFIP at a concentration of 1 mg mL^-1^ under shaking at 4 °C for 4 h in a sealed vial for complete dissolution. After that, the peptide was stored at -20 °C. Before experiments, the solvent HFIP was removed by evaporation under a gentle stream of nitrogen and then the peptide was dissolved in aggregation buffer (10 mM Tris, 150 mM NaCl, pH 7.4). The concentration of monomeric Aβ was determined by UV-Vis spectra with a calculated extinction coefficient of 1450 cm^-1^ M^-1^ at 276 nm.

**Circular Dichroism (CD) Measurements**

Aβ40 peptides (100 μM) with or without various concentrations of SeCQDs (5 μg mL^-1^ and 50 μg mL^-1^) were incubated at 37 °C for 7 days in aggregation buffer. To monitor the secondary structure changes of Aβ40, CD spectra were collected by JASCO J-1500 spectrophotometer in the range from 200 to 300 nm, with a 1 mm path length quartz cell. And the final concentration of Aβ40 used for measurements was kept at 50 μM.

**Hemolysis Experiment**

The red blood cells (RBCs) were collected from the heparin-stabilized rat blood samples via centrifugation at 3500 rpm for 10 min. Then, the remaining packed RBCs were washed with PBS (10 mM, pH 7.4) for several times to remove the plasma and buffy coat. After that, the packed RBCs were diluted with PBS in the ratio of 1:10 and put side. In hemolysis experiment, the diluted RBCs suspension (200 μL) was added to 600 μL of PBS containing SeCQDs with different concentrations. PBS and water (600 μL) were mixed with 200 μL diluted RBC suspension as negative and positive controls, respectively. All the mixtures were incubated at room temperature for 2 h. Finally, the supernatant was obtained by centrifugation and the absorbance (Abs) at 570 nm was determined by UV-Vis absorption spectra.

Hemolysis rate was calculated using the equation:

$$Hemolysis rate \% = \frac{{Abs}_{\left( Sample \right)}-{Abs}_{(Negative control)}}{{Abs}_{\left( Positive control \right)}-{Abs}_{(Negative control)}} \times100$$

**Detection of Mitochondrial Membrane Potential (MMP)**

The MMP of PC12 cells was monitored using JC-1 staining kit (Beyotime, China). JC-1 forms a red fluorescent polymer when the membrane potential is normal. In contrast, JC-1 reverse to a monomer form that emits green fluorescence in the condition of abnormal MMP. In brief, PC12 cells were plated at 50 000 cells per well on 24-well plates in fresh medium and incubated for 24 h. Then, Aβ40 (10 μM) that had been aged with or without various concentrations of SeCQDs (0.5 μg mL^-1^ and 5 μg mL^-1^) for 7 days were dispensed into the PC12 cells. After incubating for 12 h at 37 °C, the cells were stained with the JC-1 staining kit following the standard protocol. The stained cells were detected by fluorescence microscope (LEICA DMI4000B, Leica, Germany).

**Morris Water Maze Test**

Spatial memory was measured by Morris water maze test. The temperature of the room and the water was kept at 24 ± 2 °C. Before each experiment (2 h), the rats were brought to the site to allow them to be acclimated. For spatial learning, rats were trained in water maze to find a hidden platform for five consecutive days, four trials per day with a 30 min interval. Each trial was started from a predefined point (north, east, west, and south) randomly. For the probe test, the platform was removed after training for five days. The rats were allowed in the pool for 60 s. Time spent to find the platform (the escape latency) and the time spent in the target quadrant were recorded.

**Immunohistochemical Staining Procedure**

Brain samples were fixed in 4% paraformaldehyde and molded within paraffin after tissue processing. Sections of paraffin-embedded tissue (thickness: 6 μm) were prepared from CA1 area of the hippocampus. The sections were incubated with a rabbit anti-β amyloid 40 monoclonal antibody (Abcam). An immunodetection kit was used with the anti-rabbit IgG secondary antibody reacted for 1 h. Antibody staining was revealed with 3,3’-diaminobenzidine with nickel ammonium sulfate intensification. The Aβ40 labeled plaques in at least 20 fields of each brain section were counted under 40× magnification using an image analyzer.

**Nissl Staining**

Brain samples were fixed in paraformaldehyde and molded within paraffin after tissue processing. Sections of paraffin-embedded tissue (thickness: 6 μm) were prepared from CA1 area of the hippocampus. In the next step, sections were stained with 0.1% cresyl violet acetate. Five slices from the CA1 region were provided for histomorphometric analysis. The number of viable neurons was calculated and analyzed. Magnification ×40 was employed for counting the neurons, and the calculation was repeated for four fields.

**
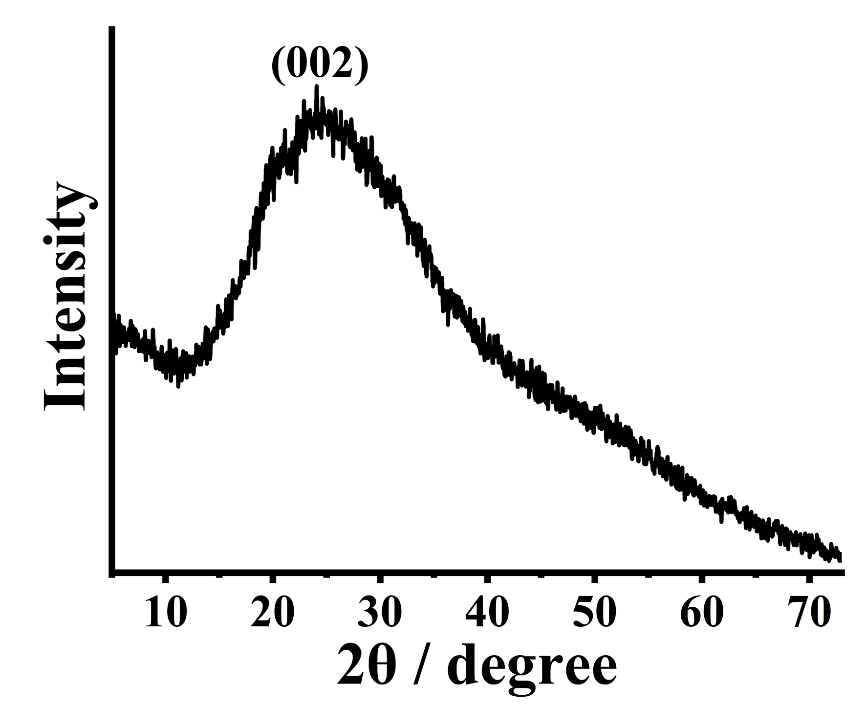
**

**Figure S1.** XRD pattern of SeCQDs.

**
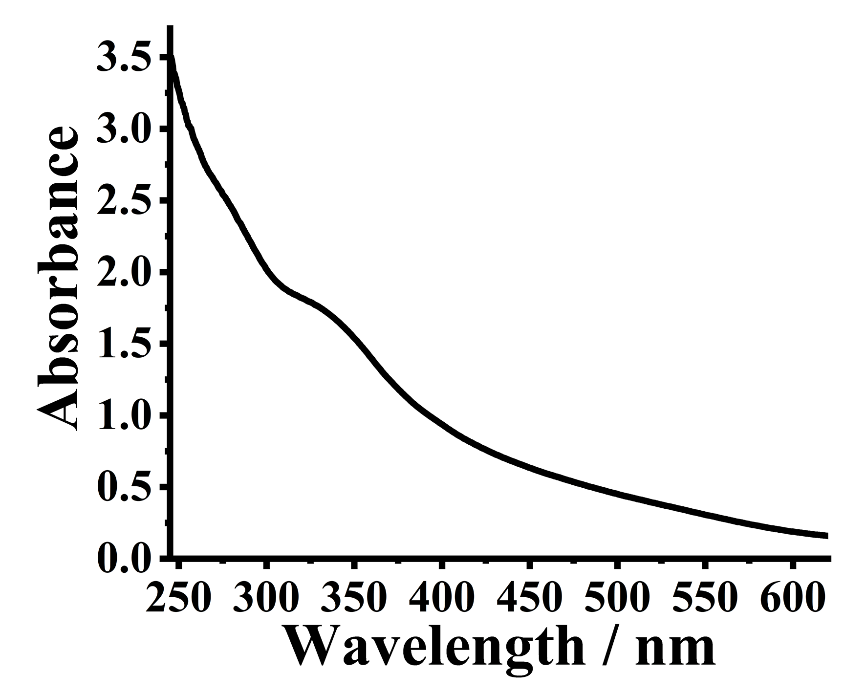
**

**Figure S2.** UV-Vis spectrum of SeCQDs.


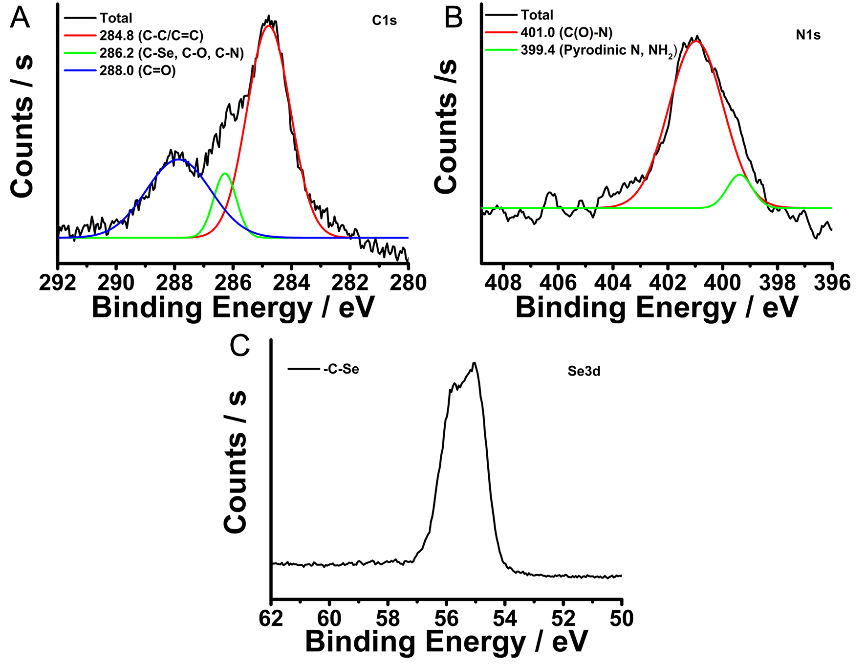


**Figure S3.** High-resolution XPS spectra of C1s **(A)**, N1s **(B)** and Se3d **(C)**.

**
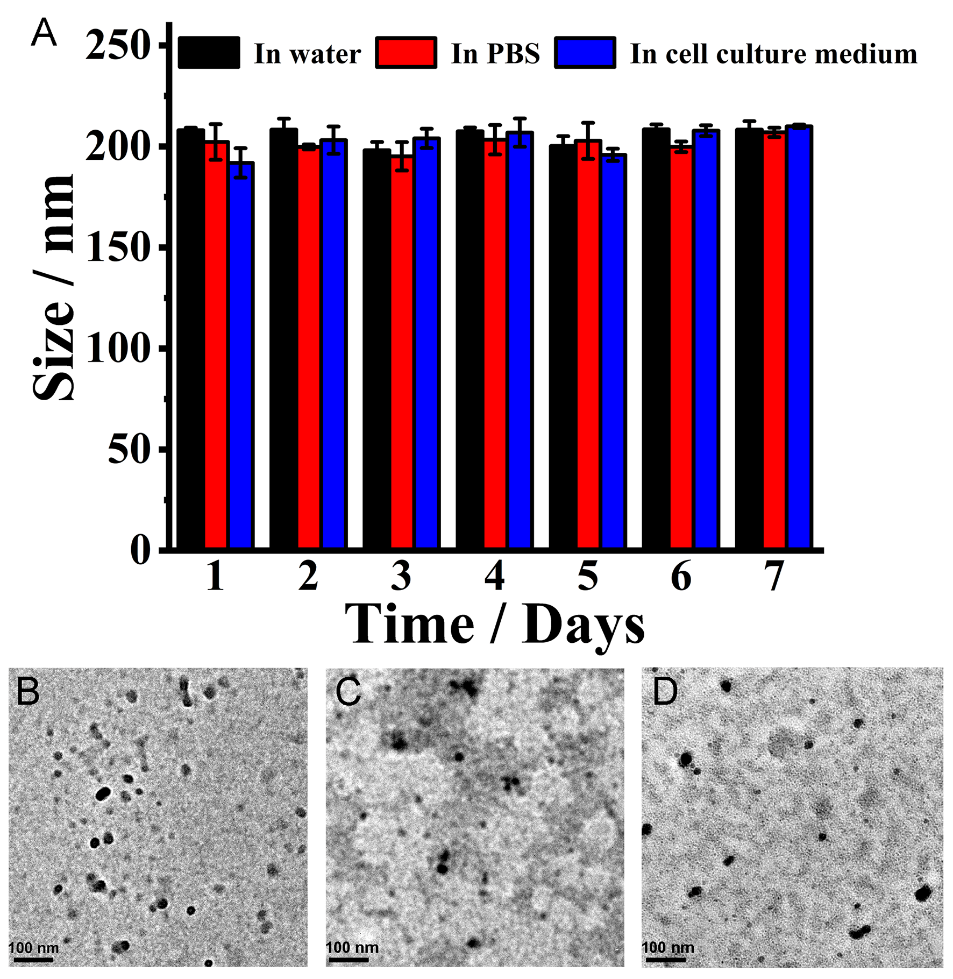
**

**Figure S4.** The stability of SeCQDs in different solutions. Dynamic light scattering studies of SeCQDs in different solutions **(A)**. The TEM images of SeCQDs incubated in water **(B)**, PBS (pH 7.4) **(C)** and cell culture medium (DMEM supplemented with 10% fetal bovine serum) **(D)** for 7 days.


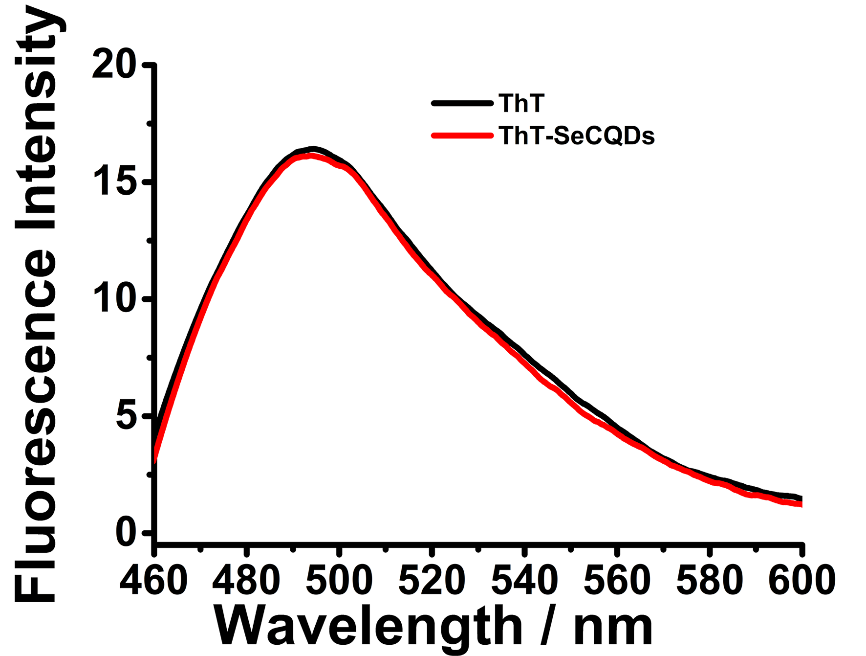


**Figure S5.** The fluorescence of ThT in the presence or absence of SeCQDs. The concentration of SeCQDs was 0.5 µg mL^-1^.


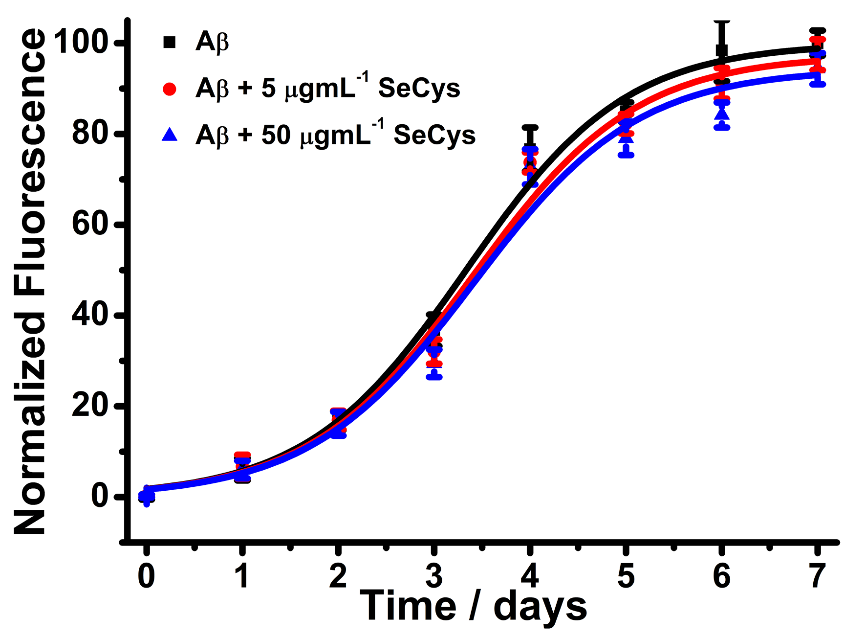


**Figure S6.** Fibrillation kinetics of Aβ40 in the absence or presence of SeCys monitored by ThT assay.


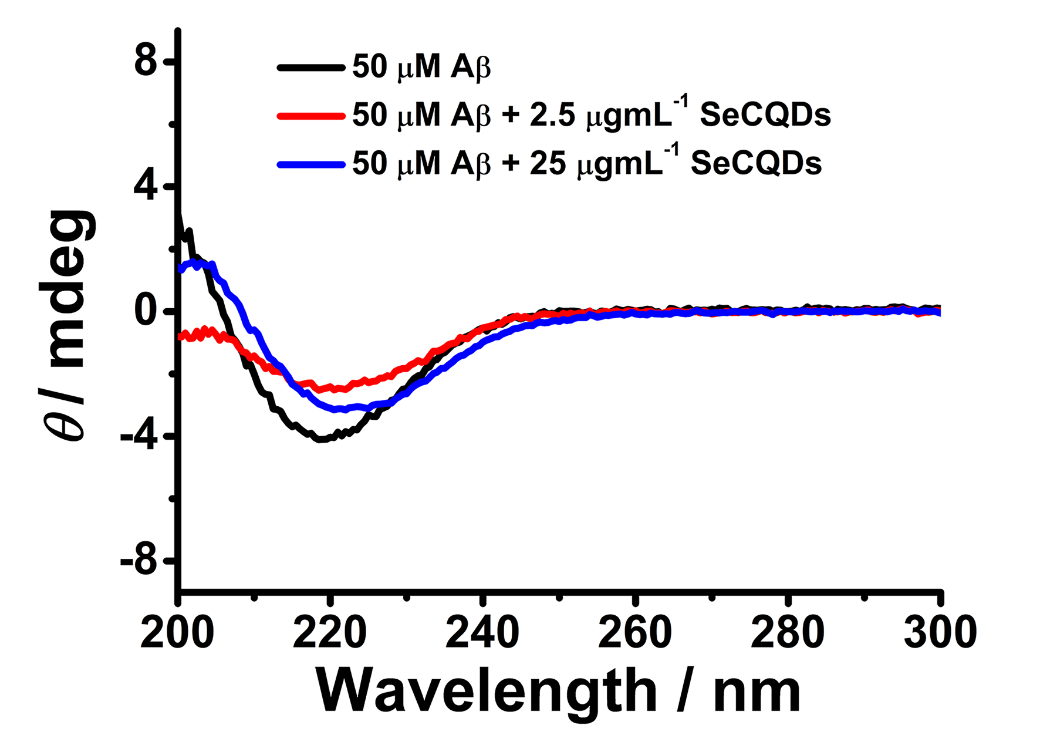


**Figure S7.** CD spectra of Aβ40 in the presence and absence of SeCQDs.


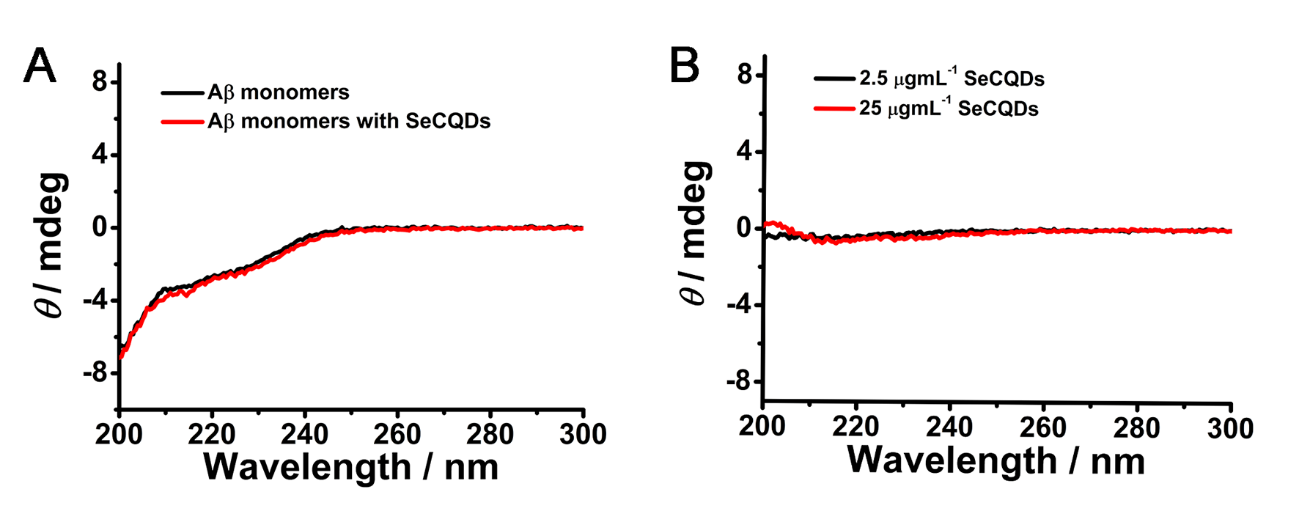


**Figure S8.** CD spectra of Aβ40 (50 µM) at the start of the experiment with or without SeCQDs (25 µg mL^-1^) **(A)** and CD spectra of SeCQDs with different concentrations **(B)**.


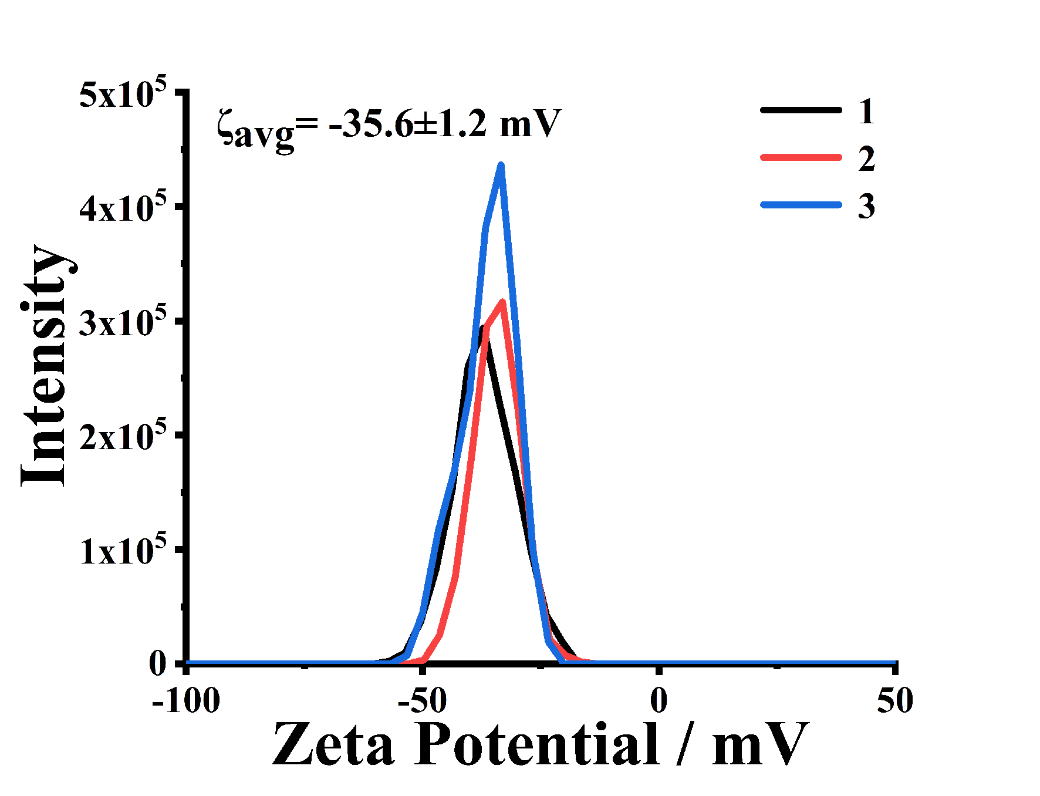


**Figure S9.** The Zeta potential of SeCQDs.


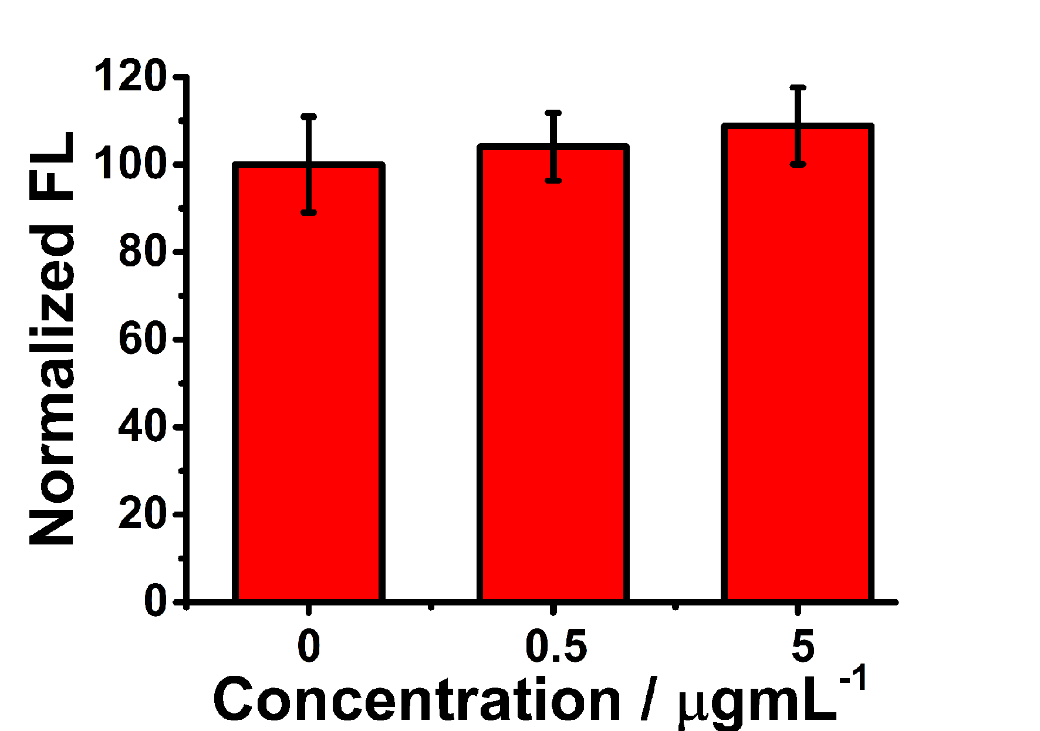


**Figure S10.** Effect of the SeCQDs on intracellular ROS formation in normal PC12 cells.


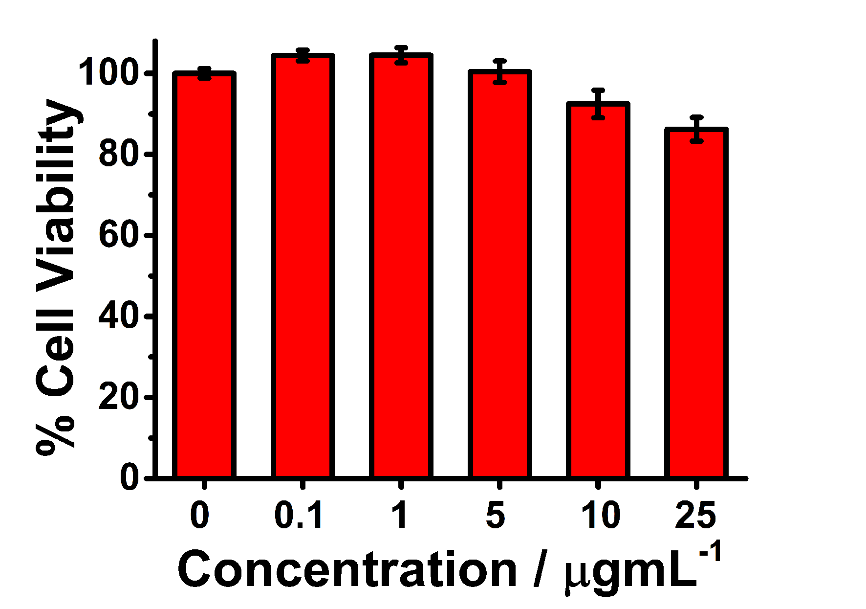


**Figure S11.** The toxicity evaluation of SeCQDs using MTT assay.


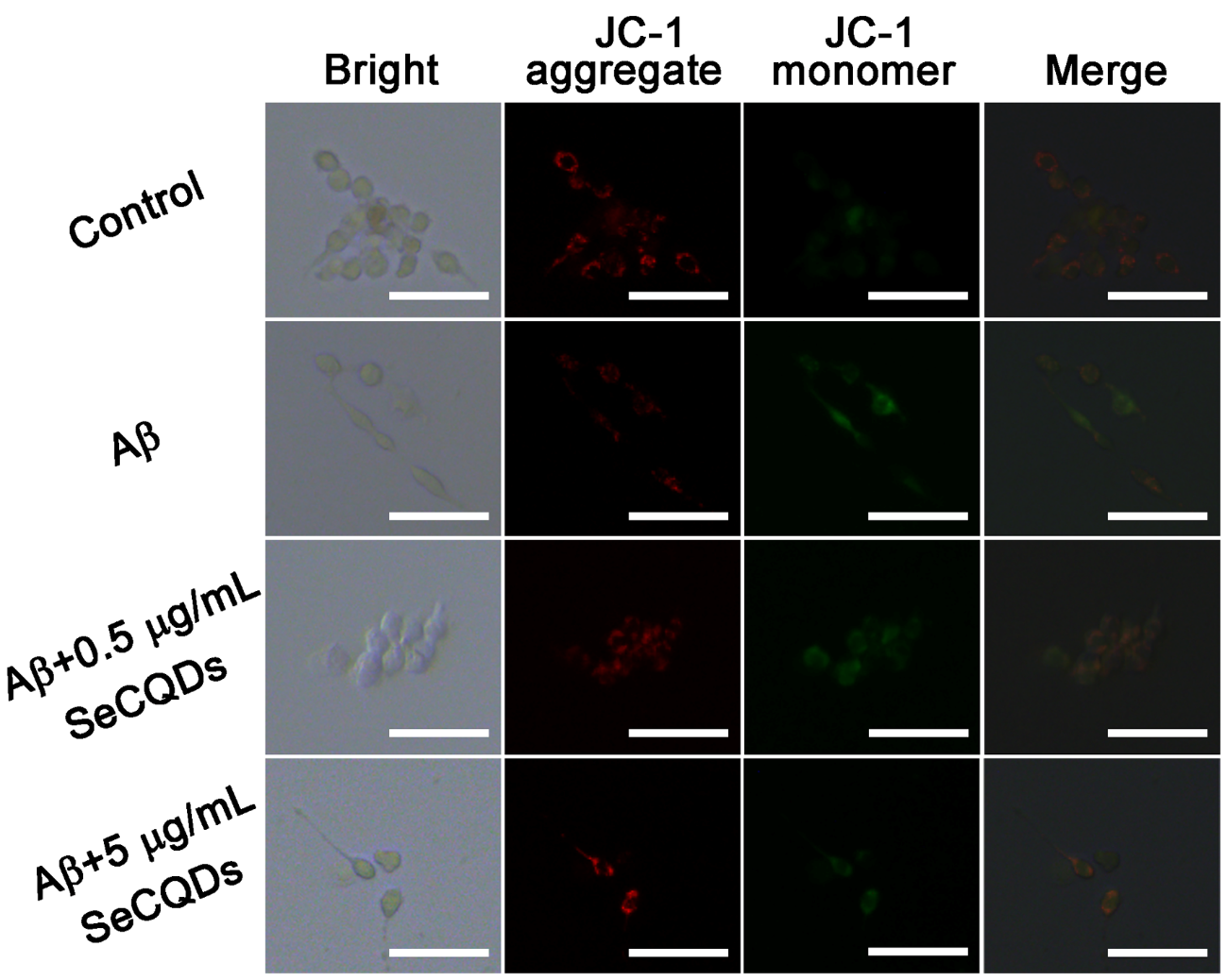


**Figure S12.** PC12 cells were stained by JC-1 to indicate the mitochondrial membrane integrity. Scale bar, 50 μm.


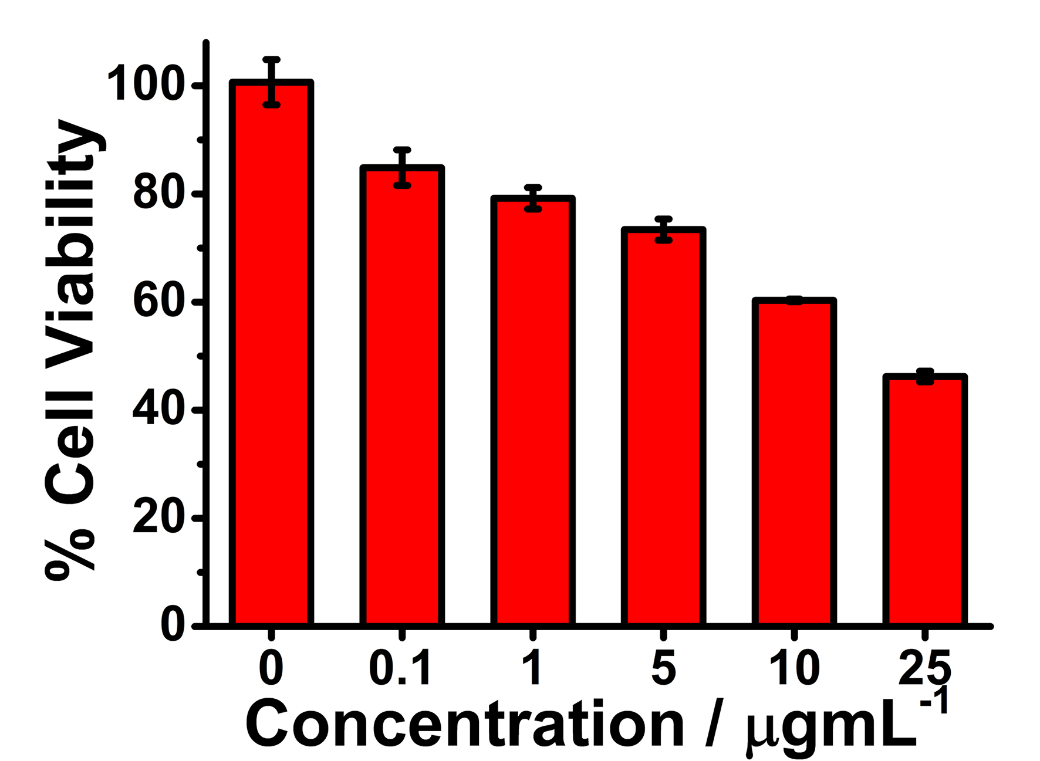


**Figure S13.** The toxicity evaluation of SeCys using MTT assay.

**References**

Kim, K., Kim, M. J., Kim, D. W., Kim, S. Y., Park, S., and Park, C. B. (2020). Clinically accurate diagnosis of Alzheimer's disease via multiplexed sensing of core biomarkers in human plasma. *Nat. Commun.* 11, 119. doi: 10.1038/s41467-019-13901-z.

Palop, J., and Mucke, L. (2010). Amyloid-β-induced neuronal dysfunction in Alzheimer's disease: from synapses toward neural networks. *Nat. Neurosci.* 13, 812-818. doi: 10.1038/nn.2583.
